# Introducing Data-Driven Materials Informatics into Undergraduate Courses through a Polymer Science Workshop

**DOI:** 10.1021/acs.jchemed.5c00562

**Published:** 2025-08-15

**Authors:** Mona Amrihesari, Blair Brettmann

**Affiliations:** † School of Chemical and Biomolecular Engineering, 1372 Georgia Institute of Technology, Atlanta, Georgia 30332, United States; ‡ School of Materials Science and Engineering, Georgia Institute of Technology, Atlanta, Georgia 30332, United States

**Keywords:** materials informatics, machine learning, polymer science, workshop, Python

## Abstract

With the rapid growth of artificial intelligence and machine learning across scientific disciplines from materials discovery to data-driven problem solving, there is increasing opportunity to integrate these tools into a broad range of applications. Successful adoption of these approaches in research can be enhanced by foundational exposure during undergraduate education. The objective of this study is to introduce fundamental machine learning concepts to undergraduate students through a hands-on, application-focused workshop during a polymer science and engineering course. Students were guided through key steps of the machine learning workflow, including data cleaning, model training, performance evaluation, and result interpretation, using a polymer solubility data set generated via visual inspection. The effectiveness of the workshop was assessed through pre- and postworkshop student surveys, which indicated a measurable improvement in students’ understanding and confidence in applying machine learning techniques. The integration of this workshop into a materials course introduces the students to the new concepts while extending the application of the course material.

## Graphical abstract

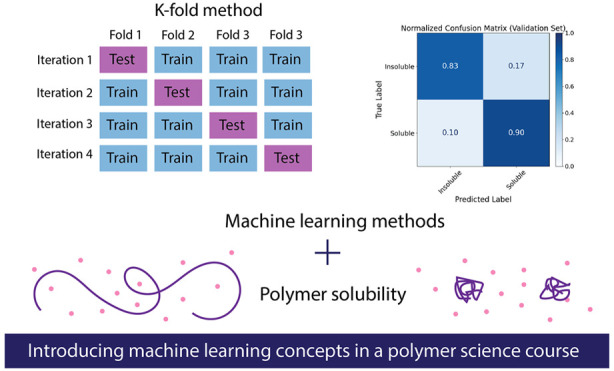

## Introduction

The discovery of new materials and further development to improve material properties are important components of chemistry, materials science, and chemical engineering research, both academic and industrial, but the process to reach these improvements is slow. Artificial intelligence (AI) and machine learning (ML) have the potential to increase the pace of materials discovery and development, particularly when large data sets are combined with AI/ML algorithms to predict properties or optimize materials design. This materials informatics approach has been demonstrated for polymer properties using Polymer Genome,^1^ advanced materials in military services,^2^ and even for industrial materials research,^3,4^ as just a few examples. Chemists and engineers will play an important role going forward in defining standards for generating data sets, collecting and documenting the data, designing ML algorithms to process the data, and interpreting and using the outputs. Thus, literacy in the basic concepts of ML for materials informatics is needed for this generation of chemists.

Although the field of AI/ML is large, if we focus on developing literacy for data-driven materials informatics for a broad range of chemists and engineers, then a few concepts come to the forefront. These include data preprocessing, types of ML models, validating and tuning the model, and how to assess the performance of the model. The first of these provides the link to experiments and involves examining a data set and using scaling algorithms to normalize the data. The second key concept is an understanding of the types of ML modeling including supervised and unsupervised, classification and regression, and some of the most important algorithms such as the decision tree or neural network. This provides important vocabulary for understanding ML studies and assessing the suitability of the input data and the type of output for the goals of a project. On the implementation side of ML, learning how to train a data set and the key decision points in validating and tuning the model breaks down the “black box” nature of the task and shows how a model can fail. Finally, metrics for assessing accuracy, precision, and recall for the ML model provide learners with tools to interpret the output of the model and assess whether the output is of sufficient quality to inform decisions in discovery and design. While other concepts are also important in AI/ML, e.g., optimization algorithms, we have focused on these as the most basic for enabling communication between those creating the model, those using the model results, and those generating the data set.

Methods for training chemists and engineers on basic AI/ML concepts for materials informatics are becoming more commonly reported. Some approaches build entire training programs, especially when the audience is graduate students specializing in ML, including the Data and Informatics Graduate Intern-traineeship: Materials at the Atomic Scale program at the University of Illinois Urbana–Champaign, the Harnessing AI for Design and Understanding Materials program at Duke University, and the AI-Enabled Molecular Engineering of Materials and Systems for Sustainability program at the University of Chicago,^5^ among others. Other approaches center around full semester courses, such as the Materials Informatics course at North Carolina State University that is the core course for an interdisciplinary graduate certificate in materials informatics.^6^ A hallmark of many of these programs is the interdisciplinary focus, which is critical for building both computational expertise and domain knowledge in materials. While these approaches provide excellent in-depth training, there is also a clear need for reaching a broader spectrum of students and providing introductory-level content.

An excellent way to build comfort and vocabulary with a new topic is through active workshops or modules that can be implemented into an existing course.^7^ This can enable a clear link between existing knowledge or course content and the new topic, improving understanding and retention while also not overwhelmingly increasing workload.^8^ In the chemical education space, multiple workshops or modules have been reported to introduce AI/ML to undergraduate students, though without a focus on materials informatics. Lafuente et al. developed a workshop (approximately 3 h of instruction, 15 h of assignments) to introduce students to Python functions, basic statistics, visualization and dimension reduction, and classification models and regression models through activities using Python Notebooks.^9^ Similar to the module we present here, the workshop focused on training in the process of using ML to analyze a data set, but it is designed as a stand-alone workshop that might be too lengthy for integration into a course. Multiple groups have developed assignments or modules that use machine learning to analyze spectra, such as those from FTIR, mass spectrometry, UV–vis, Raman, and NMR.^10–13^ These teach basic ML algorithms, classification of the data, and assessment of model performance, with a clear link to core chemistry course content through spectroscopy. In these cases, prior coding experience is not expected, and the activities are well-designed to be implemented in a short module with room to expand for students with coding experience.^9^ In all cases, student surveys and feedback showed an improved understanding of ML and the target concepts, demonstrating the ability of the workshop/module approach to improve ML literacy.

In this work, we present a module for introducing materials informatics to undergraduate students through integration with existing courses. Unlike prior efforts that focus on ML applications in spectroscopy or general chemical data analysis, this module specifically targets the application of ML to material property prediction of properties relevant in polymer science and chemical engineering separations classes. We focus on the four general topics shown in Figure 1, with specific examples selected for each topic to keep the module length to 1.5 h, though we discuss some variations that can be implemented with more time. To teach these topics, we use the example of predicting polymer solubility. In many application spaces, the solubility of a polymer in a solvent or a solvent in a polymer is important in the design of materials and processes. This includes the selection of solvents for material processing such as electrospinning and inkjet printing^14,15^ and the design of membranes.^16^ We chose this example because it integrates well into multiple undergraduate courses, including Polymer Science and Engineering and Chemical Engineering Separations, and because it is an active challenge in the field of materials informatics.^17,18^

**Figure 1:**
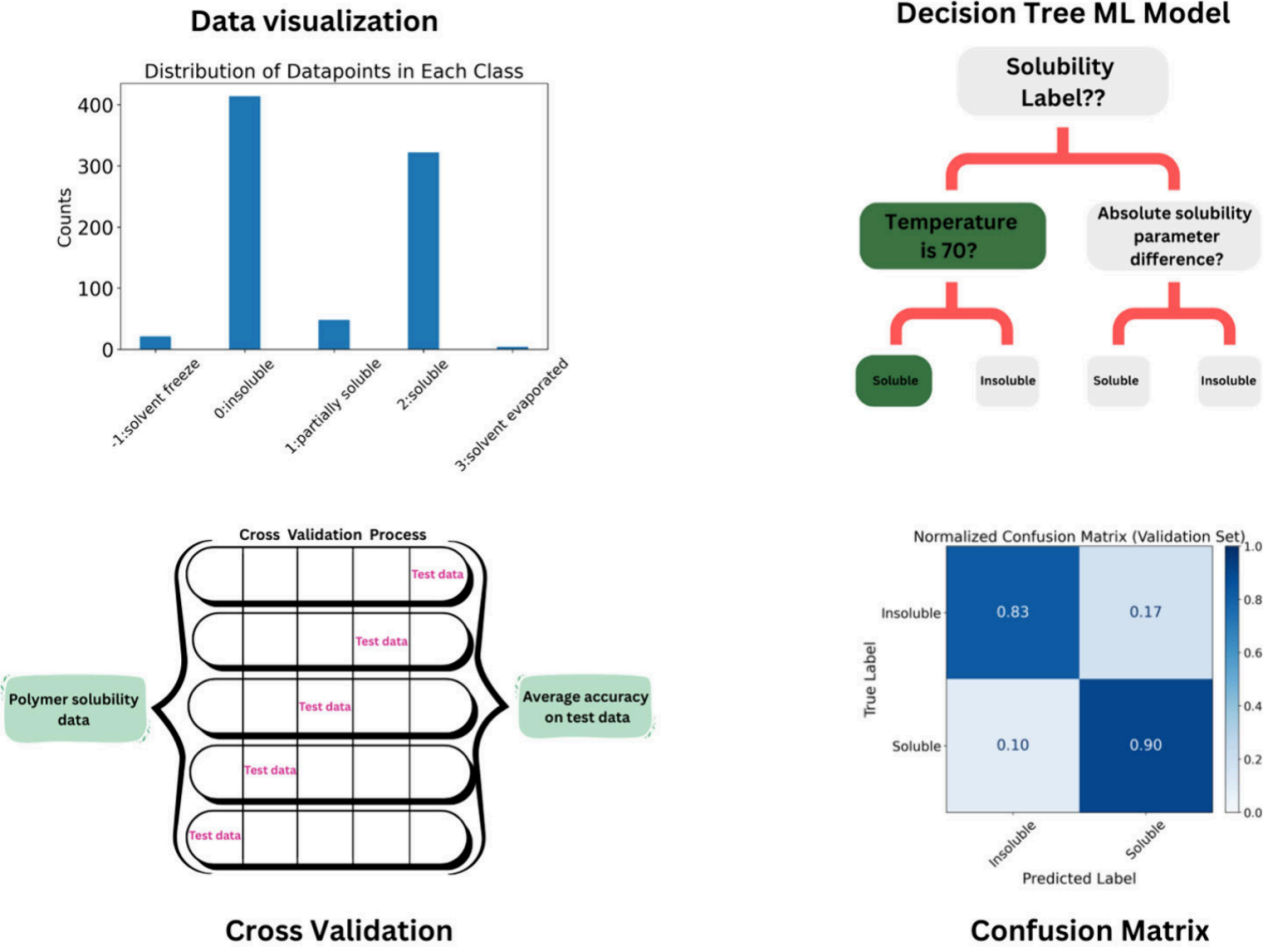
Schematic diagram illustrating the learning objectives of the workshop. The process is designed to help students understand the importance of data distribution, the need for model evaluation using techniques such as hold-out and cross-validation, the role of the model, and the performance assessment through a confusion matrix.

## Learning Objectives

This workshop is designed to equip participants with foundational knowledge and practical skills in essential machine learning techniques, specifically in the context of polymer solubility (Figure 1). Upon completion, participants will be able to

- Analyze the nature of a data set using visualization techniques.
- Explain and implement cross-validation techniques to evaluate how well a model will generalize to an independent data set, specifically
  - The hold-out method, by splitting data sets into training and testing subsets.
  - The k-fold cross-validation method, including selecting an appropriate number of folds and interpreting validation results for model evaluation.
- Understand the difference between supervised/unsupervised machine learning models and the purpose of a decision tree classifier.
- Compute and interpret a confusion matrix, including
  - Calculating and explaining the metrics of accuracy, precision, and recall.
  - Applying these metrics to assess model performance.

This set of learning objectives offers a well-rounded and accessible introduction to data science for undergraduate students, particularly those with limited or no prior experience in the field.

## Data Set

The current data set was generated to study the solubility of polymers across different temperatures in a variety of polar aprotic, polar protic, and nonpolar solvents using a visual inspection technique.^19^ This method is a qualitative approach that has been widely used by experimentalists for solvent selection. The samples were prepared using 15 unique polymers and 34 different solvents, with each sample consisting of 25 mg of polymer dissolved in 5 mL of solvent. Experiments were conducted under three temperature conditions: room temperature (25 °C), elevated temperature (65–70 °C), and low temperature (5–10 °C). For consistency in reporting, the high and low temperatures were standardized to 70 and 5 °C, respectively. A hot bath was used for the elevated condition, while a cryocooler was used for the low temperature control. Samples were stirred at 700 rpm and kept overnight at the designated temperature, with solubility observations recorded the following day. Solubility outcomes were categorized into three groups: soluble (when the solution was fully clear), partially soluble (when the solution appeared mostly clear but contained minor suspended particulates), and insoluble (when the solution was visibly cloudy or showed signs of polymer precipitation). Instances of solvent freezing and evaporation were also recorded as part of the data cleaning process demonstrated to the students. To improve consistency, a subset of experiments was independently verified by two researchers, and ambiguous cases were resolved through repetition. The distribution of solubility labels for soluble, insoluble, and partially soluble classes for different temperatures is shown in Figure 2, and an Excel sheet containing the data set is included as supplementary file “Polymer Solvent Solubility”.

**Figure 2:**
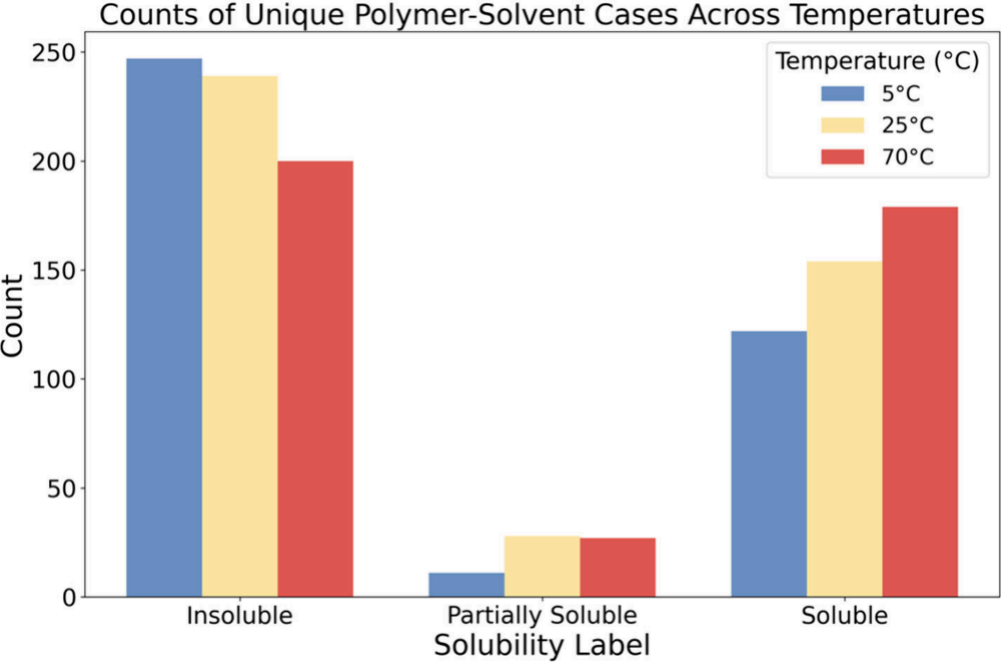
Distribution of the data points for each insoluble, soluble, and partially soluble class with respect to the temperature. The number of valid rows retained after cleaning is 809 data points, and the insoluble class has the highest number of data points. In this workshop we only focused on soluble and insoluble classes.

One of the first steps in any data-driven project is to understand the structure and quality of the data set, including the distribution of values, presence of outliers, and missing data.^20,21^ This foundational step is especially important in ML workflows, where model performance can be significantly impacted by data quality^22^ or some models are not able to handle the data sets with missing values. A key part of this process involves handling missing values, either by imputing them using strategies such as mean or median substitution (commonly applied to time series or numerical data)^23^ or by removing rows or columns containing missing entries altogether.

In this workshop, we did not delve into imputation techniques; however, students engaged with the data set through visualization and performed basic data cleaning. Specifically, they removed labels with a very low number of data points, such as “solvent freeze”, “solvent evaporated”, and “partial solubility” classes, as well as any rows containing missing values. For the data set used in this study, we focused on retaining only valid rows with complete entries. Additionally, to ensure a well-balanced data set, we concentrated on samples labeled as soluble or insoluble since these classes had a comparable number of data points and did not require advanced class-balancing methods. This step reinforces the importance of thoughtful data preparation in the building of robust and interpretable models.

Another important aspect of preparing data for machine learning models is fingerprinting, a process that enables the model to recognize and learn from the chemical interactions between polymers and solvents and relate these to the final solubility label.^24^ In this workshop, a curated set of 28 handcrafted features was added to the data set to capture relevant chemical and physical properties of the system. These features included temperature conditions, polymer and solvent molecular weights (g/mol), polymer glass transition temperature (K), solvent boiling and freezing points (K), polarity index, the absolute difference between the solubility parameters of the polymer and solvent (mPa^0.5^), and the presence or absence of specific functional groups. This feature set provided a meaningful numerical representation of each sample, allowing models to learn patterns that contribute to solubility behavior.

## Workshop design

To make the activity accessible and aligned with current industry practices, we chose to implement it using Python, the predominant language in AI and ML, due to its open-source nature, ease of use, and the wide availability of scientific libraries and built-in functions. We used Google Colab as the programming environment, which offers the advantage of not requiring software installation and allows users to run code directly in the browser, provided they have a Google account. While the activity can also be conducted in Jupyter Notebook (which requires prior environment setup), the Colab environment was preferred for its ease of access and collaborative interface, making it more effective for a classroom setting. To accommodate participants with no prior Python experience, we provided all of the necessary code along with explanations of what each section does. The primary objective was to help students interpret ML outputs and understand model behavior, not to teach programming. The notebooks were designed with editable sections where students could modify inputs (e.g., k-fold value, train-test splits ratio, or model hyperparameter) to explore how these changes affect model performance, fostering hands-on engagement with data analysis.

The activity was designed for a 90 min class period, and the overall workflow of the activity is shown in Figure 3. The instructor began with a short motivation for machine learning/data science for materials informatics that included an example of how polymer solubility predictions might be useful for membrane design. Throughout the rest of the workshop, the instructor presented for 5 min or less on a given topic, followed by ∼10 min of work time for the students to implement the code. Figure 3 shows the 10 stages of the activity, with the light colors (odd numbers) indicating times when the instructor gave short lectures and the dark colors (even numbers) indicating times when the students worked on their computers and the instructor answered questions and gave 1-on-1 feedback. This alternating style allowed the students to receive an introduction to the concepts and vocabulary in lecture format that was directly linked to the code they then worked with and the outputs they analyzed. Lecture slides are included as supplementary file “Machine Learning Polymer Solubility Activity”, and the code is included as supplementary file “Code for ML Solubility Predictions”.

**Figure 3:**
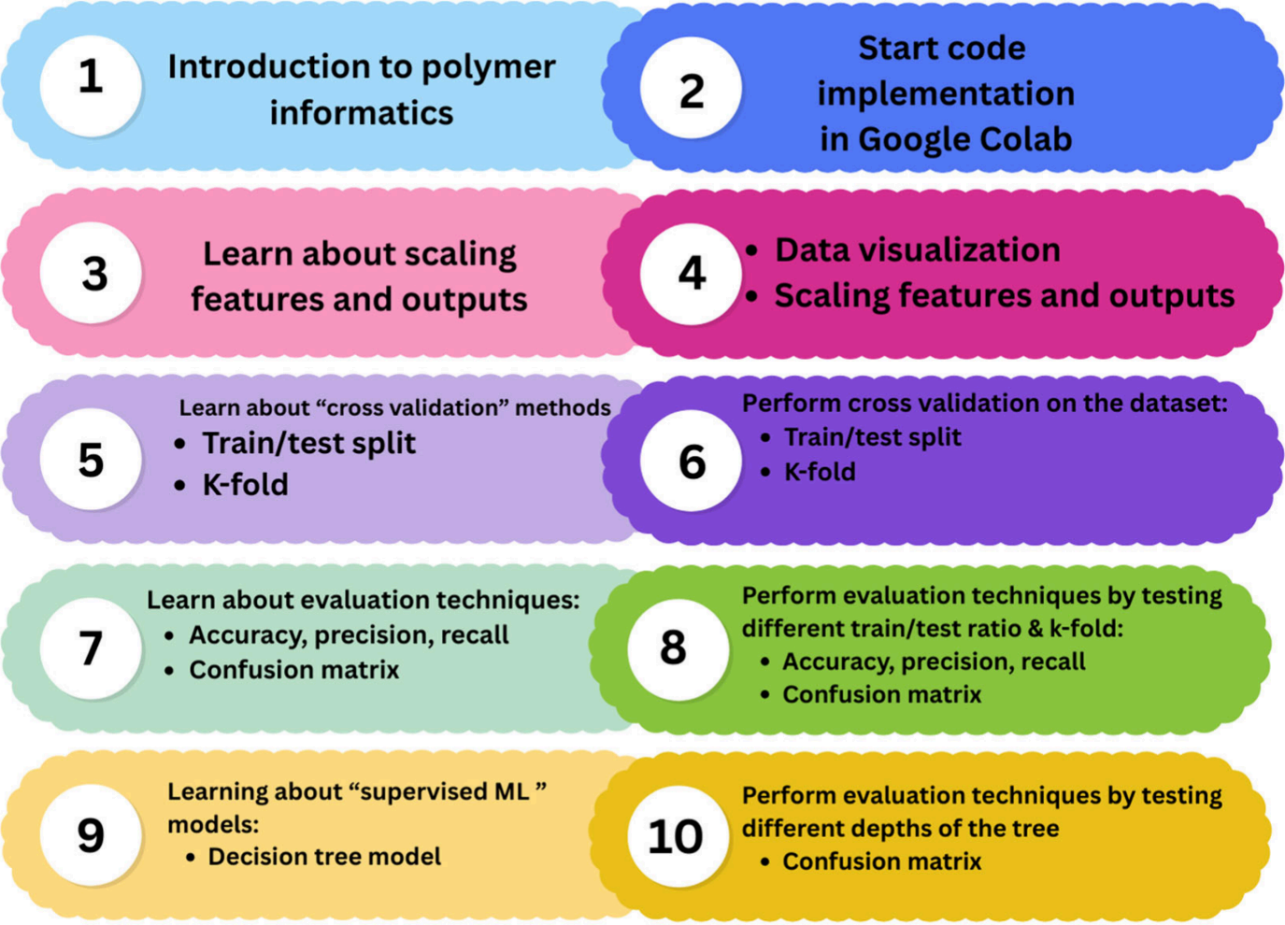
Workflow of the lecture topics and follow-up activities for the workshop. The steps show the sequential process of the lecture and hand-on activities. The thought concepts are shown in odd numbers, and activities are marked with even numbers.

As part of the workshop, students analyzed several key outputs from ML models to build their understanding of performance evaluation techniques through a guided activity sheet (included as supplementary file “ML Guided Activity Sheet”). They examined model behavior using different train-test splits (0.2, 0.3, and 0.5) and computed accuracy, precision, and recall for each case to observe how model performance changes with varying test sizes. Next, they applied k-fold cross-validation while keeping the train-test split constant—for example, at a 70/30 ratio—comparing results from 5 folds and two additional fold values of their choice to evaluate how the number of iterations affects model stability and generalization. After an introduction to decision tree models, students explored hyperparameter tuning by training models at different tree depths (3, 5, and 7) and analyzing how hyperparameter tuning such as depth influences accuracy, precision, and recall. These results were then compared to the optimal depth identified later in the activity. Finally, students generated normalized confusion matrices for the training, test, and validation sets and were asked to evaluate overall model performance on the validation set and justify which configuration yielded the most reliable predictions. These exercises encouraged students to critically analyze model behavior, compare evaluation strategies, and interpret results using meaningful classification metrics in a small scale.

## Workshop Implementation

The activity was implemented as part of a 90 min session in the Introduction to Polymer Science and Engineering course for third- and fourth-year undergraduate students. The class included 45 students, primarily from a materials science and engineering background but also including chemical and biomolecular engineering and chemistry students.

Following the structure in Figure 3, the hands-on part of the workshop started with installation of all the necessary packages including the Pandas^25^ package for loading the file, the scikit-learn^26^ package to handle the data scaling, train-test splits, ML model loading, accuracy calculation, etc., seaborn^27^ and matplotlib^28^ for visualization purposes, and the NumPy^29^ package, a mathematical computing tool. The students plotted the distribution of all the labels in the data set, as shown in Figure 4. This provided an opportunity to discuss the importance of a balanced data set on the quality of the ML model results, and as a result of the imbalance, the rest of the exercise focused only on soluble and insoluble labels.

**Figure 4:**
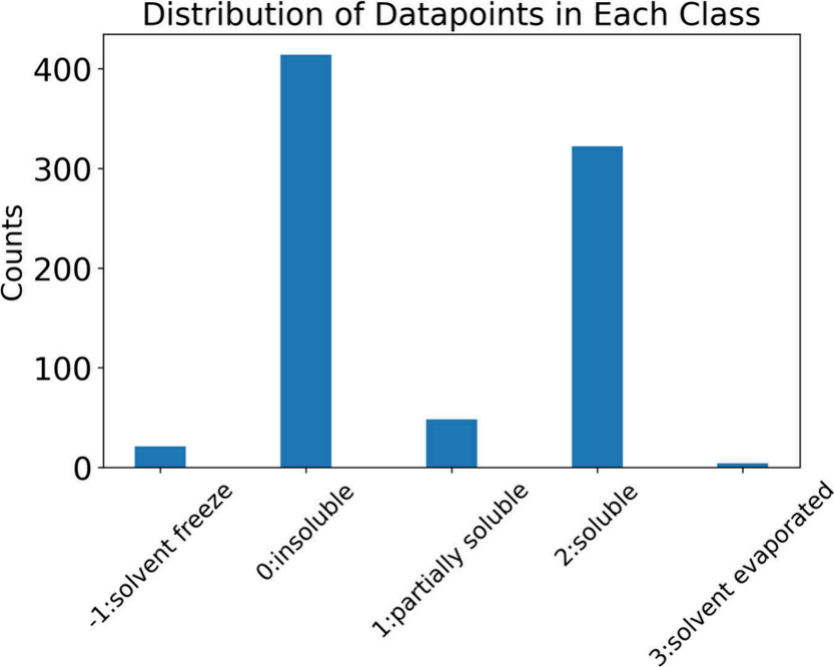
Distribution of the solubility labels in the original data set before cleaning the data points. Insoluble and soluble labels have the highest number of data points.

After cleaning the data set, the importance of feature scaling was discussed, as this step may be necessary for certain models. Although scaling is not required for decision tree models, it is essential for many statistical and distance-based methods that do not inherently interpret physical units. In such cases, skewed or unevenly scaled data can negatively impact the model performance. This concept was introduced using standardscaler from scikit-learn.^30^

Using the concepts of cross-validation,^31^ the students divided the data into two sets: a training set and a validation set. Through this activity, students learned how to implement the hold-out method using the train_test_split function, initially allocating 80% of the data for training and 20% for validation. By experimenting with additional splits, such as 70/30 and 50/50, they gained insight into how the choice of training and test set proportions can impact model performance. This helped reinforce the concept of data partitioning and allowed students to observe the trade-offs between training data availability and evaluation reliability, ultimately deepening their understanding of model generalization and overfitting in practical machine learning workflows. Once the initial split was completed, students implemented 5-fold cross-validation on the training set using the KFold method. Students were asked to further analyze different folds. To ensure reproducibility and avoid bias, the data were shuffled before splitting using shuffle = True and a fixed random_state. This iterative process helped students observe how model performance can vary across different subsets of data and reinforced the value of cross-validation for building robust and generalizable models before evaluating the final results on the held-out validation set.

Following the experience with cross-validation methods, students were introduced more thoroughly to methods of evaluating model performance, including accuracy, precision, and recall and the confusion matrix. The confusion matrix is a graphical representation of how the model predictions compare to the actual values. It has four quadrants, true positive, false positive, true negative, and false negative, and students can quickly gauge the performance of the model by examining the magnitude (visualized by depth of color) in the four quadrants, with a darker color in the quadrant indicating a greater proportion of labels in that category (Figure 5). A strong model performance will have values closer to one (deeper colors) in the true positive and true negative and values closer to zero (lighter colors) in the false positive and false negative. With this introduction to the confusion matrix, students were prepared to analyze the performance of the model.

**Figure 5:**
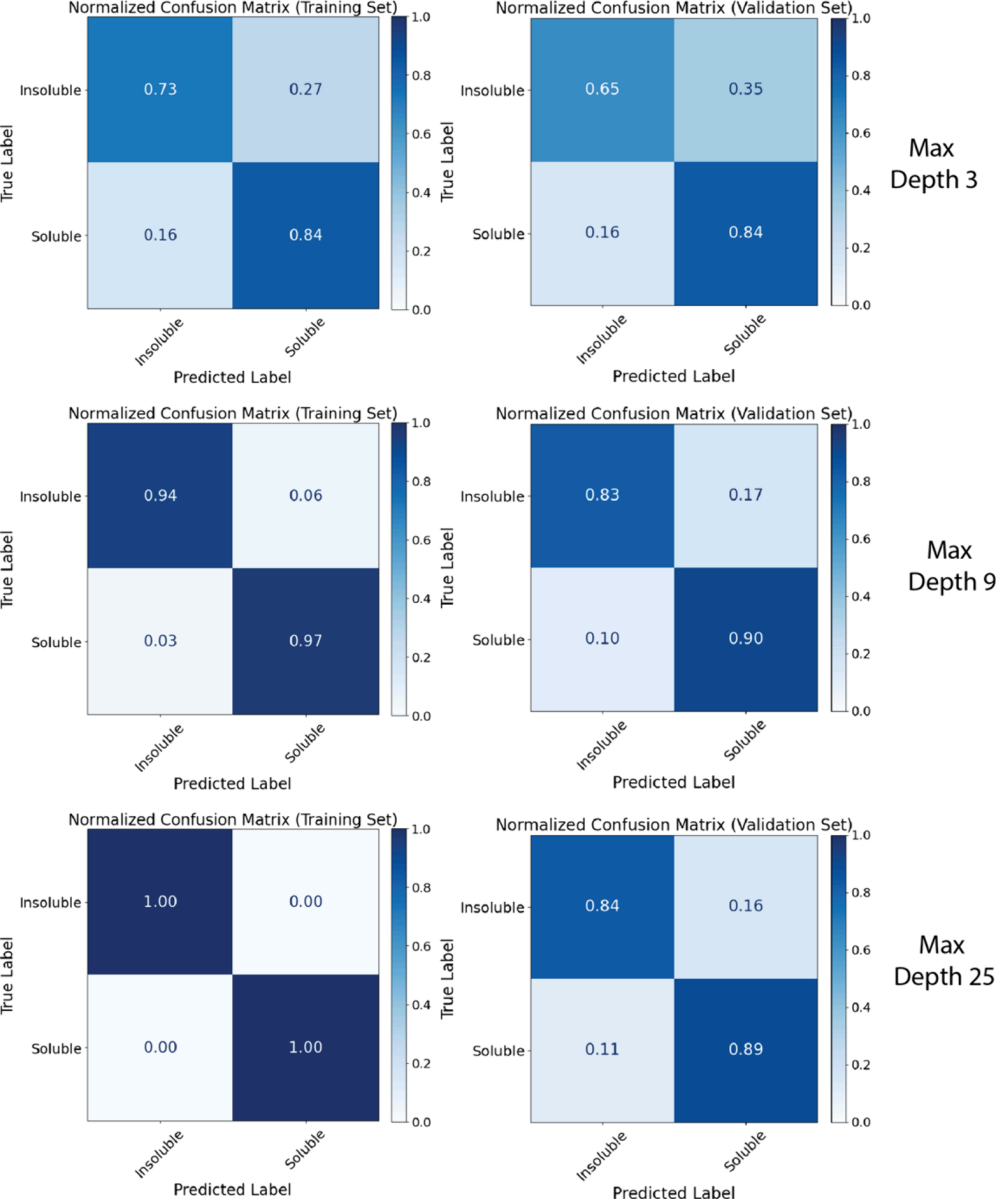
Normalized confusion matrices on the training and validation sets with train-test split at 80/20 and the k-fold = 5; max_depth of the decision tree is set to 3, 9, and 25. The students analyzed and interpreted the results for different depths of the decision tree.

While several models were explored during module development, including k-nearest neighbors (kNN), Naive Bayes (NB), Random Forest (RF), and Linear Discriminant Analysis (LDA), the decision tree (DT) model was ultimately selected for its pedagogical clarity and interpretability. A decision tree is a common type of machine learning model that uses if/then statements to reach a decision, similar to how humans think. To teach the students about the complexity of the model, we included an exercise on hyperparameter tuning. Hyperparameters are structural parameters of the model that control how it learns from the data. For our tuning exercise, the students optimized the depth of the decision tree model, which is the number of splits or nodes allowed and sets the complexity of the model (a lower number means a simpler model). This is an important parameter because allowing too much complexity can lead to overfitting, where the model essentially memorizes the data and thus cannot readily predict data that it has not seen before. However, using a decision tree model that is too simple can lead to underfitting and a lack of learning. Thus, this is a good test parameter to help the students learn the concepts of overfitting and underfitting.

By analyzing the normalized confusion matrices for each data set split, students were able to identify patterns of overfitting and underfitting. In Figure 5, the confusion matrices are shown for the training sets (left side) and test sets (right side) at 3 different decision tree depths. The max_depth of 9 is close to the optimum, and from the confusion matrices one can see that it performs better than the max_depth of 3 for the training and test set. Additionally, the max_depth of 25 shows a perfect prediction for the training set, providing an example of overfitting. By selecting an optimized tree depth of 9 to balance performance and generalization, students learned how to tune this hyperparameter to avoid overfitting while maintaining a strong predictive accuracy. This reinforced the importance of model evaluation of multiple data subsets and demonstrated how confusion matrices can be used as a diagnostic tool for informed decision-making in ML workflows.

## Outcomes and Feedback

Two surveys, pre- and postworkshop, were designed and completed by 38 students to evaluate the student feedback on the core concepts of the workshop. The questions are listed in Table 1. This set of questions is designed to assess students’ self-reported understanding, familiarity, and interest related to core concepts introduced in the workshop, as well as their perception of the relevance of data science in materials science. Questions 1–5 specifically evaluate students’ conceptual grasp of ML fundamentals, including understanding the term “Machine Learning”, common training methods, model evaluation techniques, and specific tools such as decision tree and confusion matrices. These questions gauge the effectiveness of the instructional content and activities in the building of technical knowledge. In contrast, Questions 6 and 7 focus on students’ interest and perceived value of computational tools, particularly in the context of polymer science and materials informatics. Together, these questions provide insight into both the learning-oriented outcomes and the interest-related impact of the workshop, informing instructors about areas of strength and opportunities for further engagement.

**Table 1:** Questions for Pre- and Postsurveys Submitted by the Students to Evaluate the Learning-Oriented Outcomes and the Interest-Related Impact of the Workshop

Question 1	I understand the term “Machine Learning”
Question 2	I understand some methods for training machine learning models
Question 3	I understand how to evaluate how well a machine learning model has made predictions
Question 4	I am familiar with a decision tree model
Question 5	I am familiar with a confusion matrix
Question 6	I am interested in the application of computational methods to predict polymer properties
Question 7	I think materials informatics approaches are important for materials design

The survey results for the first five questions are compiled in Figure 6. The questions were designed using a Likert scale, with 0 representing “strongly disagree”, 3 indicating “neither agree nor disagree”, and 5 representing “strongly agree”. The preassessment responses are shown in purple, and postassessment responses are shown in green. As shown in Figure 6, the workshop significantly improved students’ confidence in key machine learning concepts, including training a decision tree model and evaluating results with a confusion matrix (Questions 2–5), even among those with no prior coding experience, suggesting the code explanations and guided format were effective for beginners. Further analysis revealed that although students’ overall understanding of the term “Machine Learning” (Question 1) did not drastically improve, likely due to prior familiarity, their grasp of how to train an ML model (Question 2) showed significant gains. After the workshop, 97% of participants agreed or strongly agreed with some methods for training ML models. Questions 3 and 5 focused on evaluating the model performance using accuracy metrics and the confusion matrix. Results showed a 75.6% increase in the number of students who understood what a confusion matrix is following the workshop. Question 4, which assessed familiarity with decision tree models, indicated that more than half of the students were unfamiliar with the concept before the workshop. As highlighted in Figure 6, the workshop was highly effective in introducing and clarifying the concept of decision tree models.

**Figure 6:**
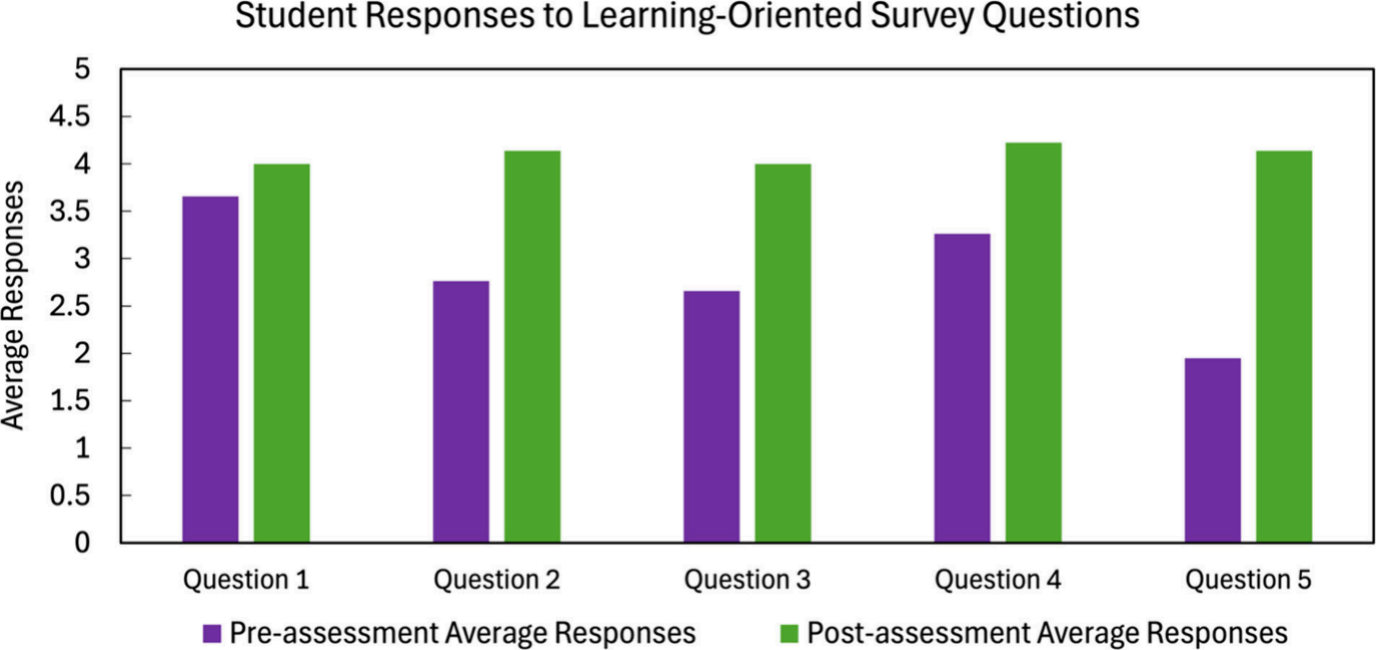
Student survey results for pre/postassessment average responses for Questions 1 to 5 focused on the learning objectives of the workshop. Question 1: I understand the term “Machine Learning”. Question 2: I understand some methods for training machine learning models. Question 3: I understand how to evaluate how well a machine learning model has made predictions. Question 4: I am familiar with a decision tree model. Question 5: I am familiar with a confusion matrix.

Figure 7 shows the results of the interest-related questions pre- and postworkshop. Although 94% of students agree that materials informatics approaches are important for materials design, the overall interest in the application of computational methods to predict polymer properties slightly increased. There is more overall certainty on the importance of materials informatics approaches in materials design (Question 7), which also increased after the workshop.

**Figure 7:**
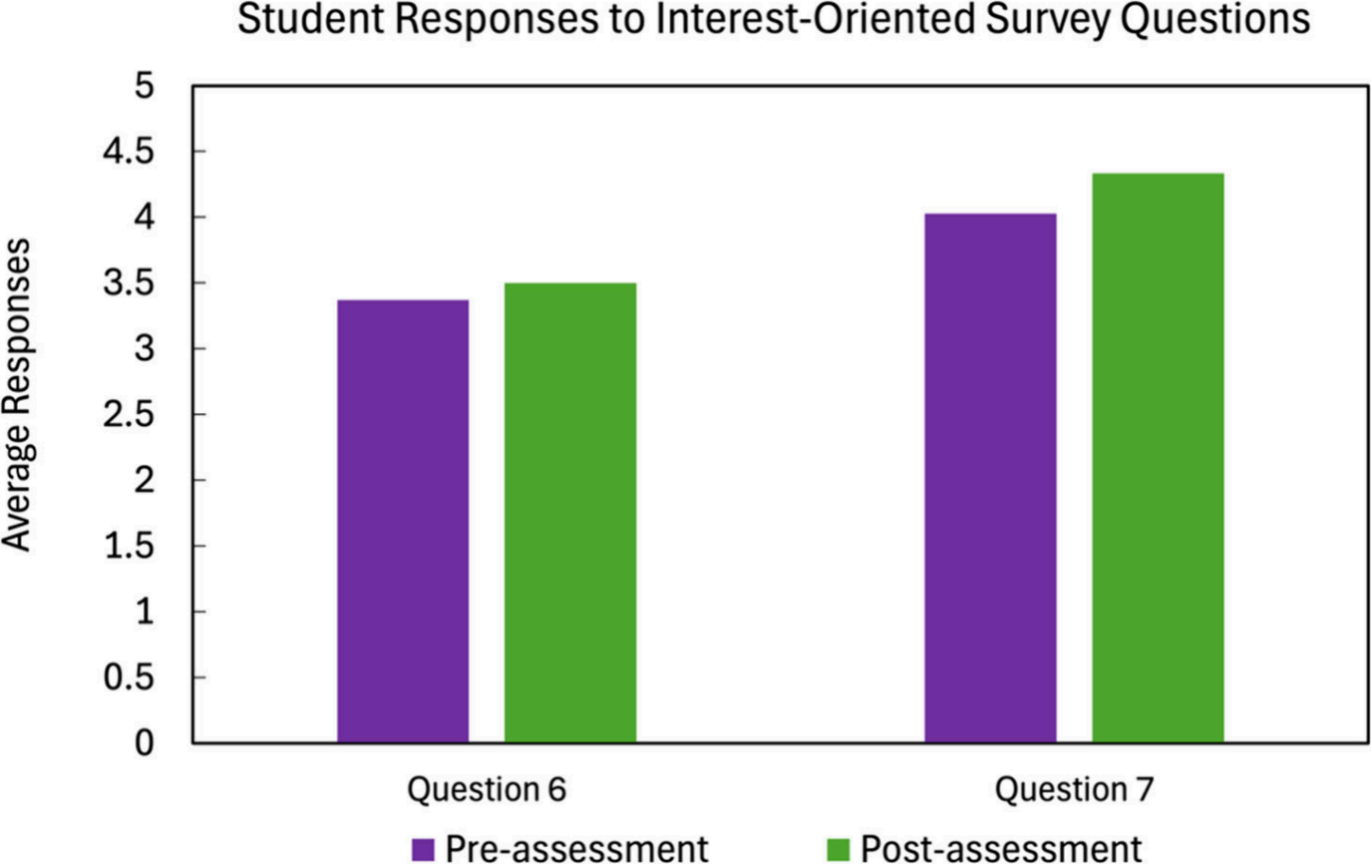
Student survey results for pre/postassessment average responses for Questions 6 and 7 focused on the interest-oriented questions on using data science in polymer informatics. Question 6: I am interested in the application of computational methods to predict polymer properties. Question 7: I think materials informatics approaches are important for materials design.

The selected questions are closely aligned with the key learning objectives of the session, enabling a meaningful evaluation of whether the intended outcomes were achieved. Questions 1 through 3 directly reflect the workshop’s goals of introducing students to supervised learning and evaluation strategies such as hold-out validation and k-fold cross-validation. Questions 4 and 5 assess familiarity with the decision tree model and confusion matrices, which were central to the hands-on activity. The significant improvement in responses to these questions indicates that students gained both conceptual and practical understanding in these areas. Finally, Questions 6 and 7 address broader objectives related to cultivating student interest in applying computational methods within materials science, particularly in predicting polymer properties using data-driven approaches. The positive responses to these questions suggest that the workshop not only conveyed technical content effectively but also increased student engagement with the field of materials informatics. Together, the alignment between the survey responses and learning objectives demonstrates that the workshop successfully met its instructional goals. In the future, a follow-up postassessment would be appropriate for tracking student enrollment in related data science or machine learning courses to further evaluate the workshop’s effectiveness in fostering interest in materials informatics.

## Variations on the Workshop

As presented here, the workshop was designed to be integrated into an undergraduate polymer science and engineering class. However, the concept of polymer solubility is applicable more broadly, and the style of the workshop is readily adaptable. In this section, we briefly discuss two variations performed with this workshop material, one in a core chemical engineering course, Separation Processes, and the other as a stand-alone workshop at a polymer science research institute. This highlights the flexibility of the material and aims to seed new ideas for educators.

Membrane separations are a growing technology for chemical processes, and the design of new membrane materials to improve separations is an active area of research. The permeability of species through membranes depends on the solubility and the diffusivity of the species in the membrane material; therefore, if these properties could be predicted, there is an opportunity for improving the design of new polymers for membrane separations. Examples of the use of materials informatics for this purpose include works by Lee et al.^18^ and Wang et al.,^32^ among others. Given this close link between membrane separations and polymer solubility, we implemented this activity in Separation Processes, a third-year undergraduate class in the School of Chemical and Biomolecular Engineering. We piloted two styles for the workshop: one nearly identical to that described for the polymer science class but with more discussion of membrane separations and the second as a series of two lectures with the implementation and analysis of the code performed as a small group project.

In this second iteration, with the two lectures and group project, we went into more depth in the lectures on each of the topics described above and added significantly more content on types of ML models to the lectures, including both regression and classification models. We also expanded the analysis beyond solubility to include glass transition temperatures, melting points, decomposition temperatures, densities, cohesive energy densities, fractional free volumes, tensile strengths, and Young’s moduli. For this, we provided an additional data set with these features and had the students determine which features had a linear relationship with O_2_/N_2_ selectivity. This expanded assignment enabled students to implement ML regression models and analyze their performance, all in the context of the course content for membrane separations. The structure of lectures and group project received mixed feedback, as some students found it to be too difficult given their unfamiliarity with Python, while others found it to lack sufficient depth for a project. Thus, we chose to perform all work in class in further iterations, though we could also integrate an introduction to Python earlier in the class to provide a foundation to make it a project with greater depth.

The second variation of this workshop was performed with researchers at the Institute of Macromolecular Chemistry at the Czech Academy of Sciences in Prague, Czech Republic, which hosts the UNESCO/IUPAC Postgraduate Course. The participants consisted of approximately 15 students and career researchers working in macromolecular chemistry. In this variation, we introduced the topics with a series of talks: the first focused on a fundamental study of polymer solubility, and the second focused on using ML to predict polymer solubility. We then implemented the workshop as described for the polymer science and engineering class. As these were more experienced participants, there were more in-depth questions during the session and good discussion on how this might be used in various research projects. Importantly, there was significant interest in available data sets and how to find or generate appropriate data sets. From this experience and feedback we received, we think this workshop is also a good introduction for researchers, though more content on applications and data sets would enhance it further for this audience. These examples demonstrate that the workshop structure is flexible and can be adapted to a variety of educational contexts, ranging from undergraduate courses to research training, with adjustments to the course level, class size, and discipline, supporting its broader generalizability across institutions.

To support instructors in implementing the workshop, we developed a comprehensive set of teaching materials. These include (i) annotated Jupyter Notebooks with line-by-line explanations (“Code for ML Solubility Predictions”), (ii) presentation slides summarizing key ML concepts (“Machine Learning Polymer Solubility Activity”), and (iii) an instructor guide outlining workshop structure and expected outcomes with a suggested grading rubric (“ML Guided Activity Sheet”). These resources are intended to reduce the barrier to adoption for instructors who may not have prior experience with Python or machine learning.

## Conclusion

We presented a workshop design to introduce undergraduate students to concepts in ML/AI during a core course, polymer science and engineering. The workshop is designed for 90 min and introduces the topics of analyzing the nature of a data set, cross-validation methods, the decision tree classifier, and the confusion matrix. Based on surveys before and after the activity, we show that student understanding of basic ML concepts improves significantly and that their level of interest improves modestly. We also discussed two additional implementations of the workshop, in a Separation Processes course and as a stand-alone workshop at a research center, showing the versatility of the program. Use of this and similar short workshops has the potential to build some foundational knowledge for students on ML/AI topics important for materials design while tying it well to existing curriculum content.

## Supplementary Material













## Notes

Polymer Solvent Solubility: Data set of polymer/solvent solubility (XLSX)
Machine Learning Polymer Solubility Activity: Lecture slides to accompany activity (PowerPoint version available upon request) (PDF)
Code for ML Solubility Predictions: Code for the students to implement during the activity (PDF, DOCX)
ML Guided Activity Sheet: Assignment sheet provided with the activity (PDF, DOCX)
