# Supplementary material for: Introducing Data-Driven Materials Informatics into Undergraduate Courses through a Polymer Science Workshop
Source: J Chem Educ. 2025 Aug 15;102(9):3972–81. doi: 10.1021/acs.jchemed.5c00562 (PMC12424157; doi:10.1021/acs.jchemed.5c00562)
Supplement: Supplementary file 2 [file ed5c00562_si_002.pdf]

Supporting Information

Introducing data-driven materials informatics into undergraduate courses through a polymer science workshop

Mona Amrihesari,[1] Blair Brettmann[1,2*]

1) School of Chemical and Biomolecular Engineering, Georgia Institute of Technology, Atlanta, GA 30332
2) School of Material Science and Engineering, Georgia Institute of Technology, Atlanta, GA 30332

*corresponding author, blair.brettmann@chbe.gatech.edu

Teaching slides

Content begins on next page

Polymer property prediction through machine learning/ data science

Workshop module designed by Mona Amrihesari
And Blair Brettmann

Machine learning/ data science for materials inf[illegible]

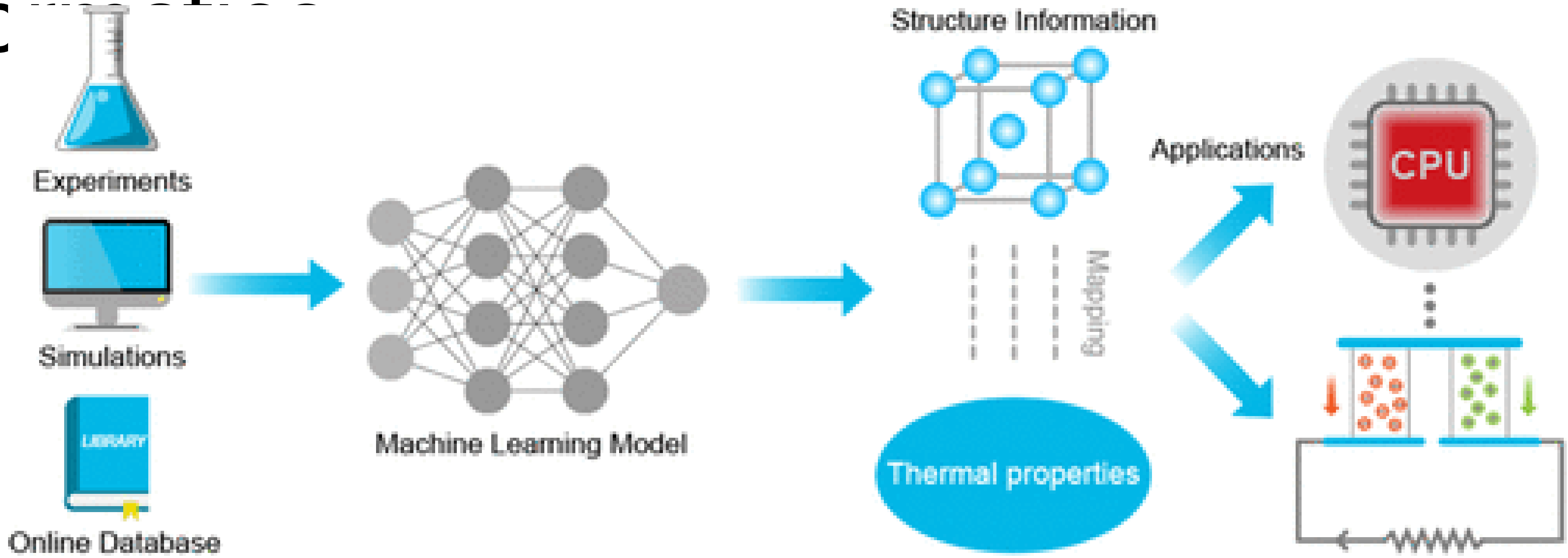

Figure from Wan et al. Nano Letters 2019, DOI: 10.1021/acs.nanolett.8b05196

- Create databases through experiments, simulations, literature extraction
- Train machine learning model to link structural features to properties
- Predict properties with model

Example: Data science to predict polymer membrane properties

- For dense membranes the permeability for each species depends on the:
  - Solubility of the species in the membrane
  - Diffusivity of the species through the membrane
- To design better membranes, can we predict polymer structures that optimize solubility/diffusivity of each species for the best separation?

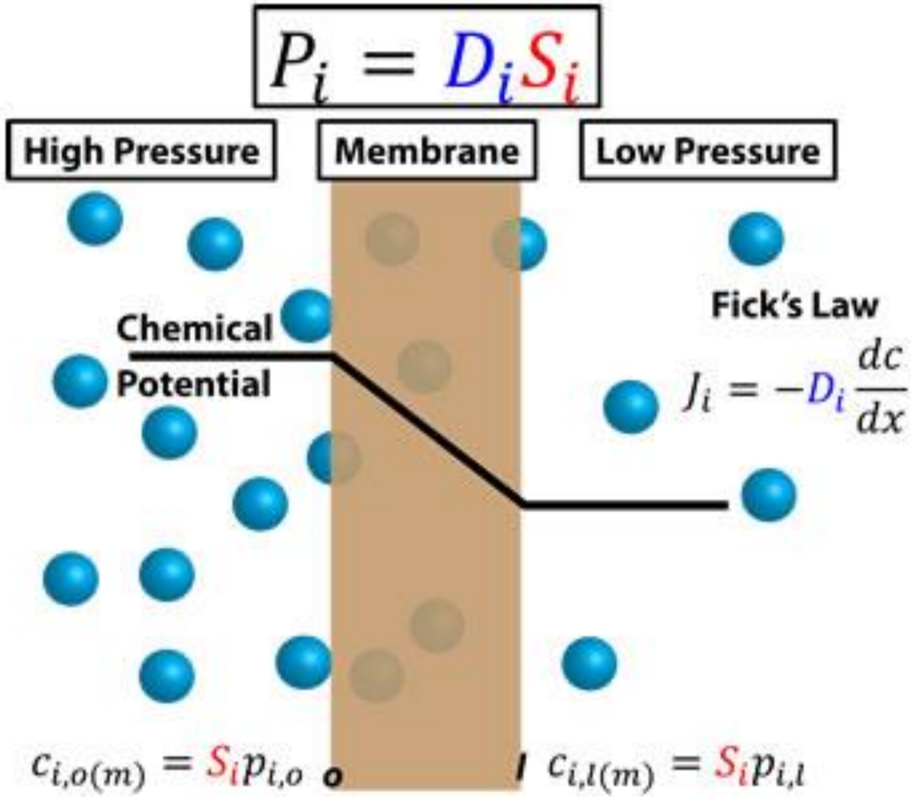

Figure from: Zachary Smith webpage, https://smithlab.mit.edu/research/

Evaluating the performance of the model

- How well is a machine learning model performing?
- Potential questions:
  - How well does it learn from your data to predict properties that *were in the training data set*?
  - How well does it learn from your data to predict properties that *were NOT in the training data set*?
  - How well do the results represent ”real” properties
    - Garbage in – garbage out

Getting started

- https://colab.research.google.com
- Press on “New Notebook” button
- Now, press on the files on the left sid
- (Polymer _solvent_solubility.xlsx)

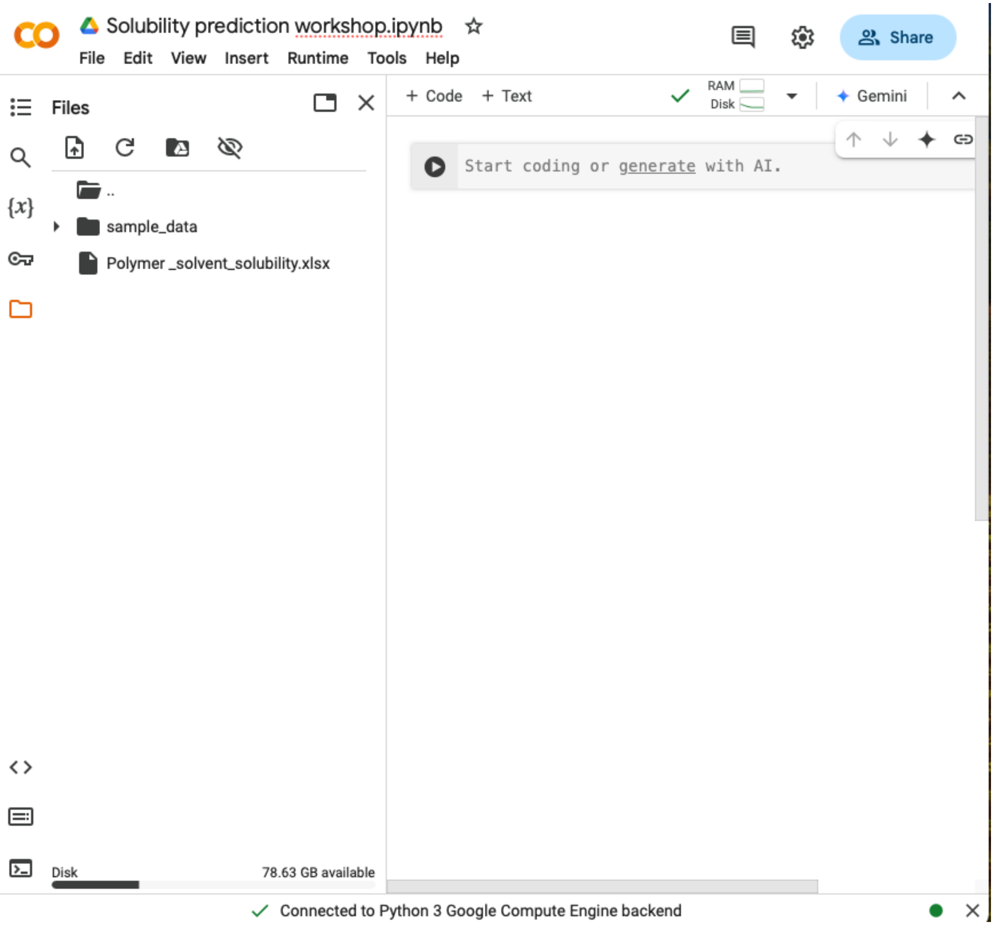

Getting started

- General coding in google collab:
  - Add code to the boxes (use the same grouping as in the code document)
  - Press play
- Set up code and import/prepare data for activity (follow instructions from code document through step 8)

Look at the data – is it well-balanced?

Topics for today

- Scaling features and outputs
- Cross validation methods
  - Train/test split
  - K-fold
- Evaluating model performance
  - Accuracy, precision, recall
  - Confusion matrix
- Decision tree classifier

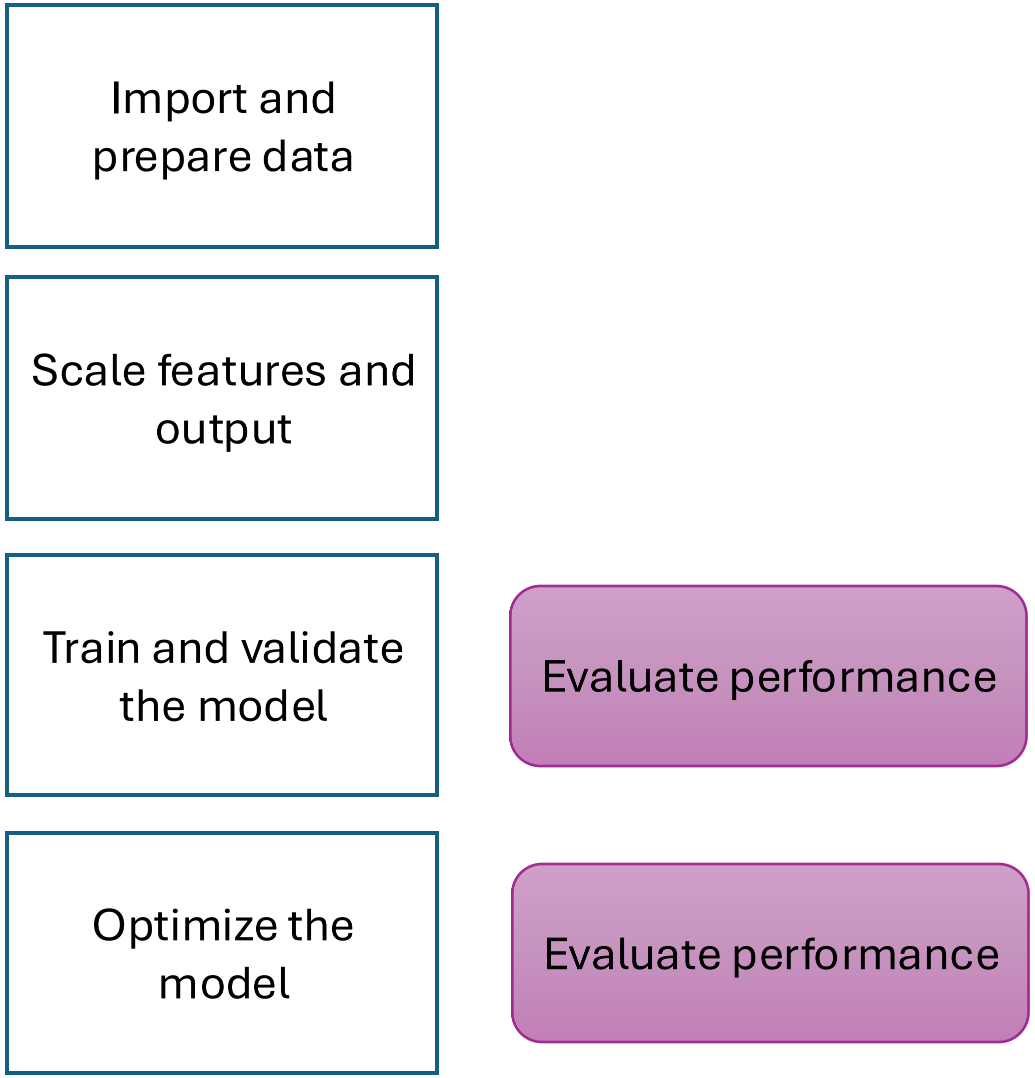

Scaling features and outputs:

- Skewed data and outliers can negatively impact the performance, so we may need to scale the features
- Statistical methods don't know about physical units, so we can normalize or "scale" features to aid in comparison:

  - rescaling: 0 = min, 1 = max
  - mean scaling: 0 = mean, 1 = max, -1 = min
  - **standard scaling: 0 = mean, 1 = standard deviation**
  - unit vector: the length of each multi-dimensional vector is 1

```
ss = StandardScaler()
X_ss = ss.fit_transform(X)
y = y.astype(int)
```

Step 9 in code document

Topics for today

- Scaling features and outputs
- Cross validation methods
  - Train/test split
  - K-fold
- Evaluating model performance
  - Accuracy, precision, recall
  - Confusion matrix
- Decision tree classifier

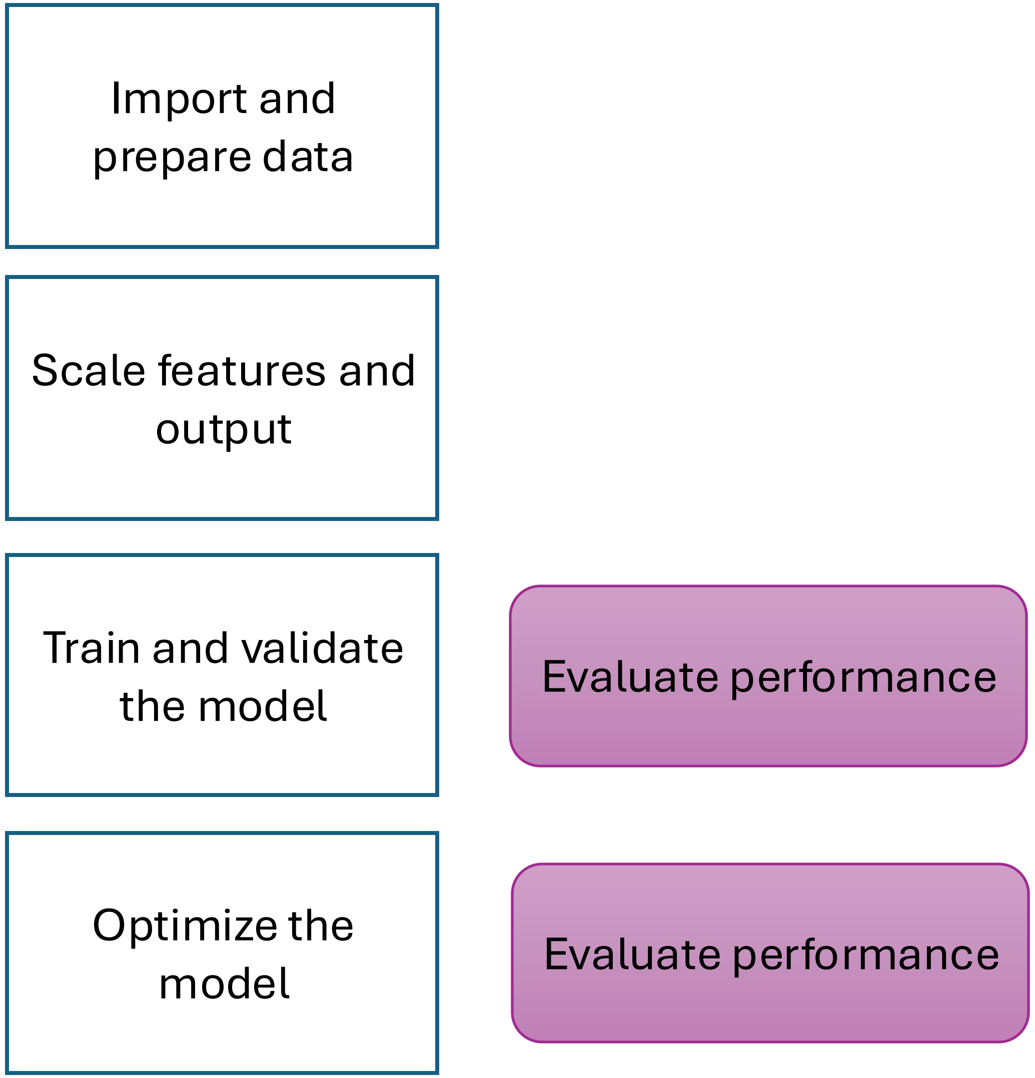

Cross validation

- Evaluate the performance of a ML model by training the model on subsets of data and then evaluating them on additional subsets of data
- Key evaluation points:
  - **Overfitting**: The model has memorized all the training data and will be perfect on the training data and terrible on testing data
  - **Underfitting**: The model is "guessing" at the data and will be equally bad at predicting the data it has been trained on and the data it is tested on

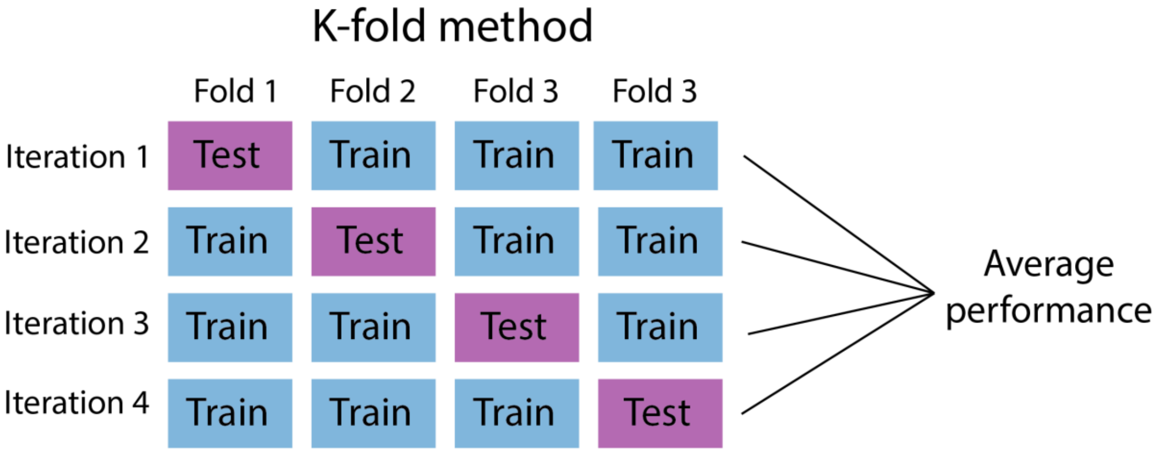

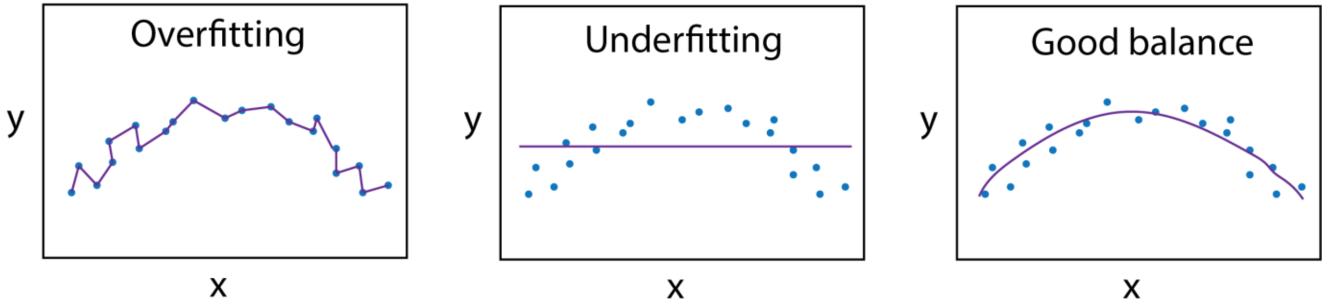

Train/test split: Hold out method

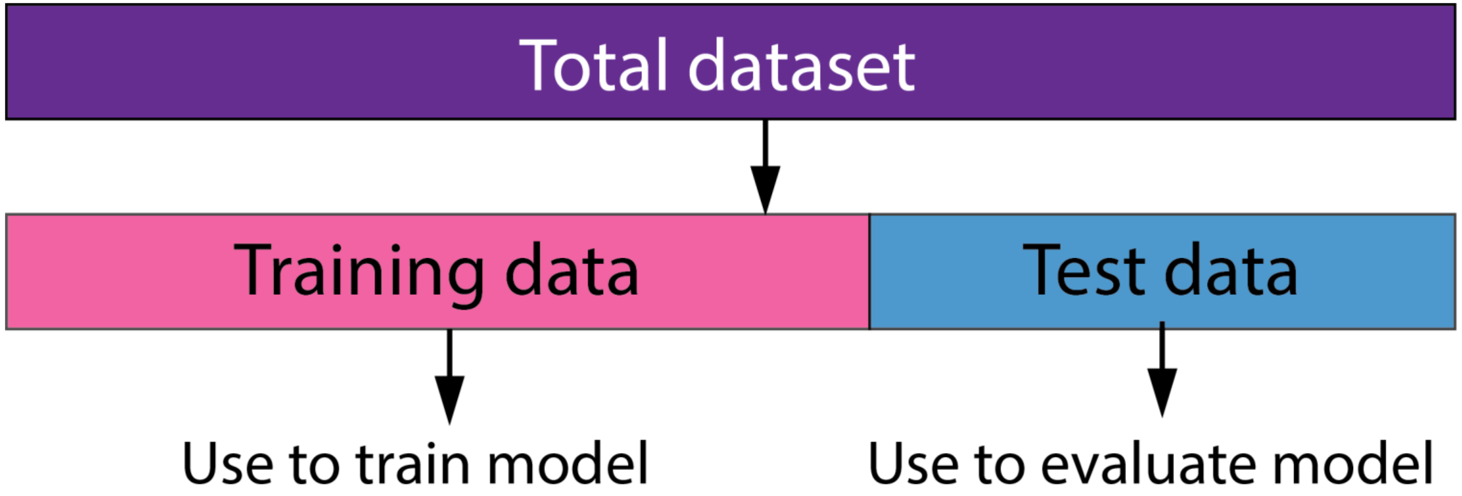

- Split the data into training and testing sets, evaluate the model performance
- Evaluate based on mean absolute error (or similar) between predicted and actual data in the test set

Train/test split: Hold out method

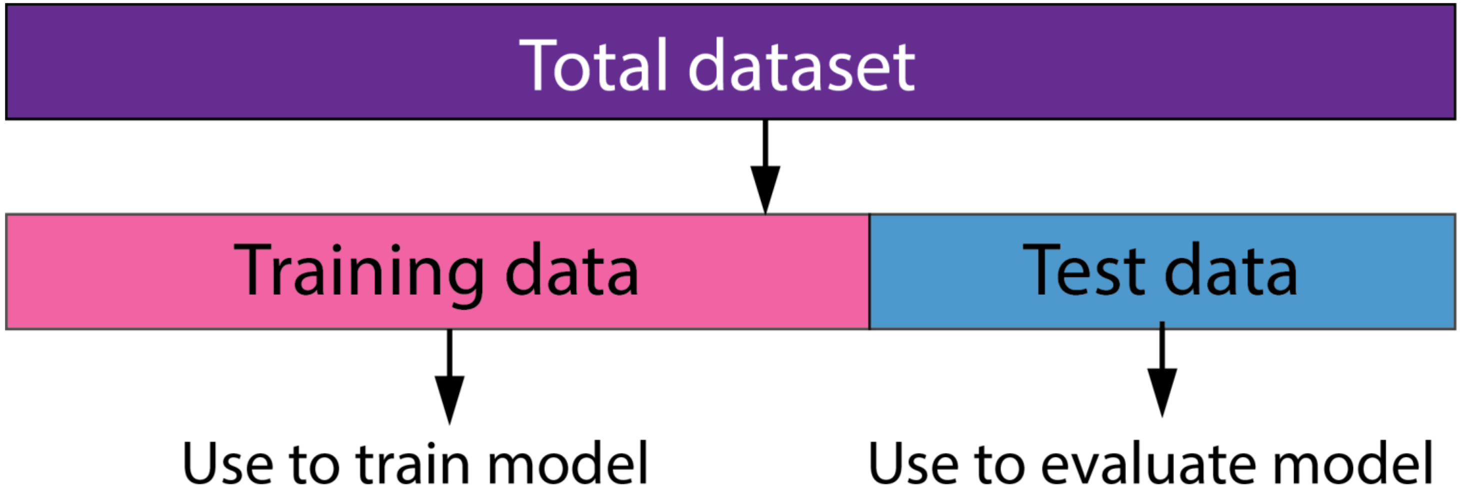

- The measured error can be highly variable. It depends on which observations end up in the training set and test set
- This is a particularly big problem when you don't have large data sets, as it can lead to overestimation of the error

Code for train/test split for hold out method

```
X_train, x_validation, Y_train, y_validation =
train_test_split(X_ss, y, test_size=0.3, random_state=42)
```

Step 10 in word document

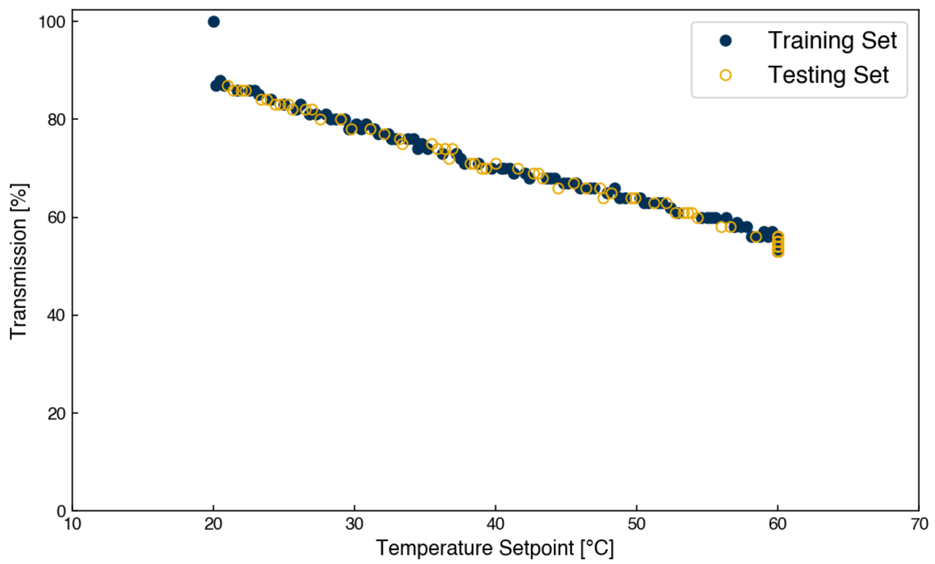

- train_test_split easily performs hold-out.
- If you change the test size, you can see how withholding different amounts of data impacts the training of the model

K-fold method

- Divide the data into k subgroups randomly
- Train the model k times, holding each group out once
- Less variation in error because you average over multiple runs of the model
- But tak[illegible] accura[illegible]

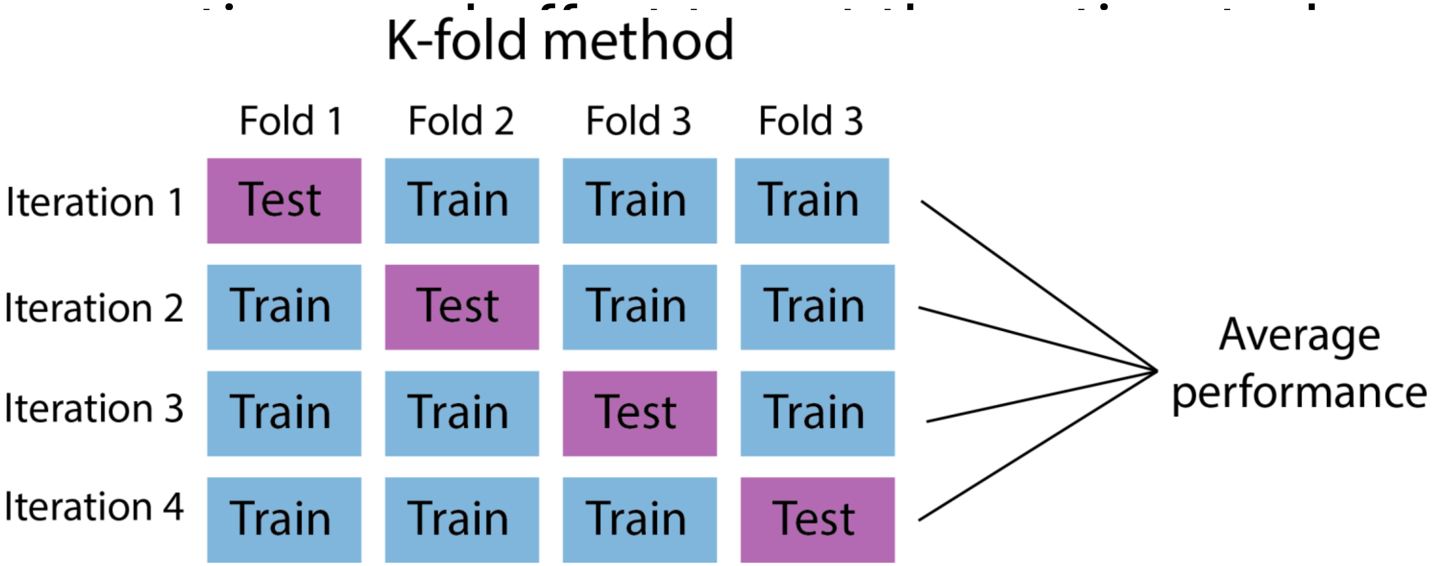

Code for K-fold method

```
kf = KFold(n_splits=5, shuffle=True, random_state=42)

for train_index, test_index in kf.split(X_train):
   x_train, x_test = X_train[train_index], X_train[test_index]
   y_train, y_test = Y_train[train_index], Y_train[test_index]
```

Step 11 in word document

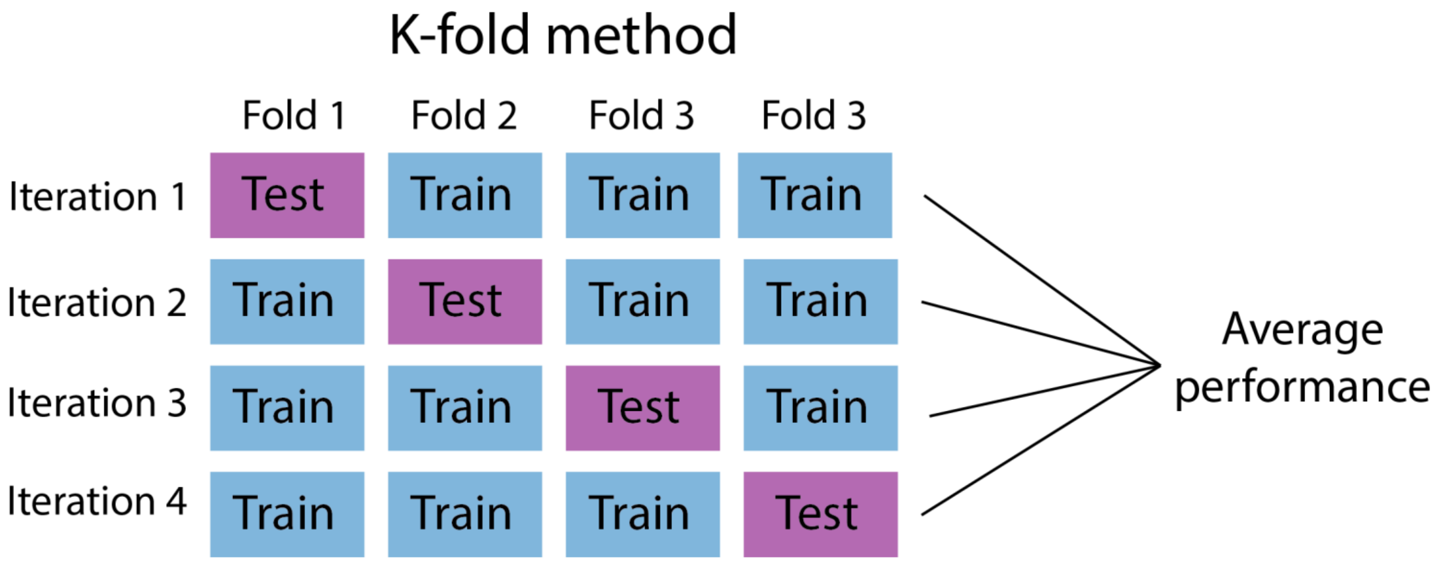

Topics for today

- Scaling features and outputs
- Cross validation methods
  - Train/test split
  - K-fold
- Evaluating model performance
  - Accuracy, precision, recall
  - Confusion matrix
- Decision tree classifier

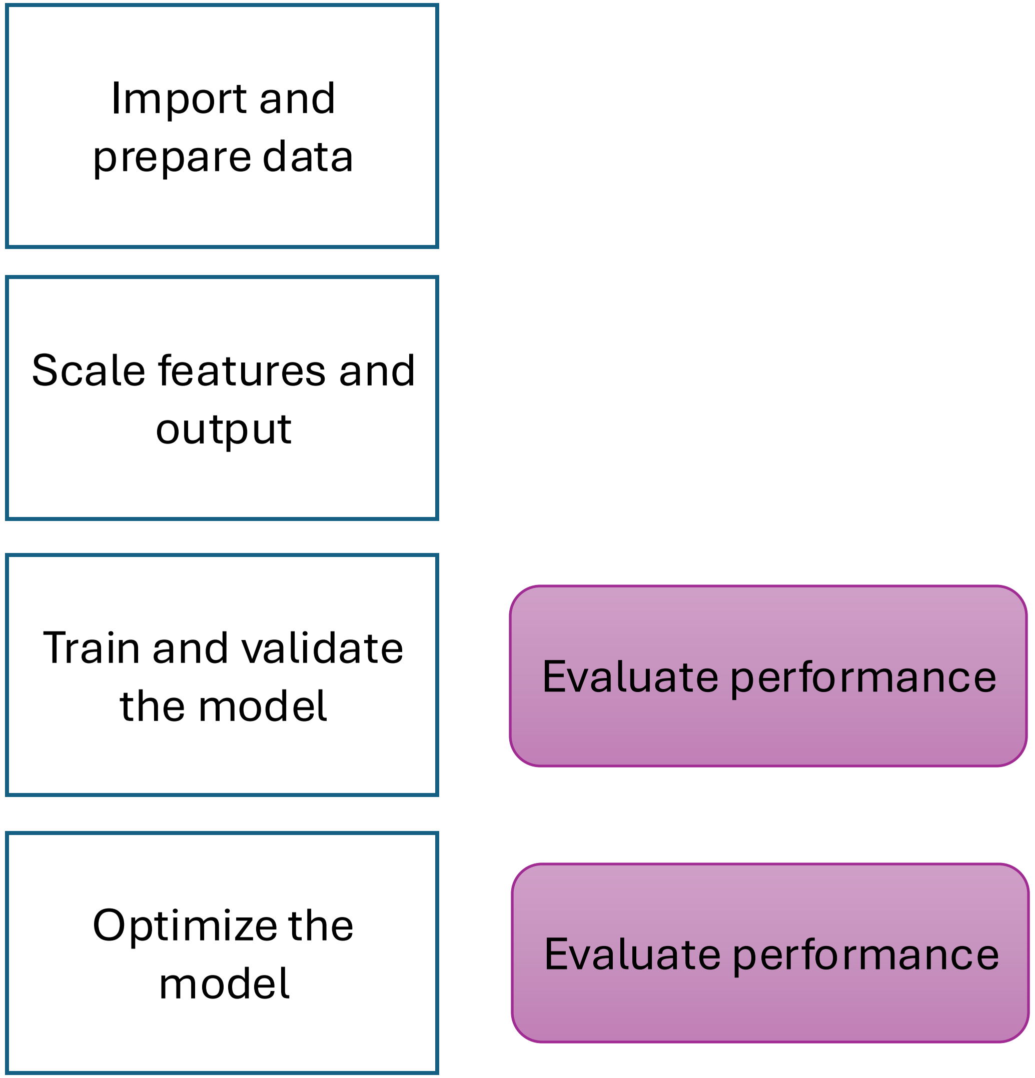

Accuracy, Precision, Recall:

3 common metrics for evaluating 2-class models:

- Accuracy
- Precision
- Recall

Accuracy

$$\frac{TP + TN}{TP + TN + FP + FN}$$

The proportion of all classifications that were correct

Precision

$$\frac{TP}{TP + FP}$$

The proportion of the positive classifications that are actually positive

Recall

$$\frac{TP}{TP + FN}$$

The proporotion of actual positives that were classified correctly as positive

TP = True positive
TN = True negative

FP = False positive
FN = False negative

Calculation of accuracy, precision, recall

```
y_validation_pred = dt.predict(x_validation)

# Calculate metrics with zero_division handling
accuracy = accuracy_score(y_validation, y_validation_pred)
precision = precision_score(y_validation, y_validation_pred,
average='weighted')
recall = recall_score(y_validation, y_validation_pred,
average='weighted')

print("Accuracy:", accuracy)
print("Precision:", precision)
print("Recall:", recall)
```

Step 13 in word document

Accuracy

$$\frac{TP + TN}{TP + TN + FP + FN}$$

Precision

$$\frac{TP}{TP + FP}$$

Recall

$$\frac{TP}{TP + FN}$$

The Confusion Matrix

Predicted label		Ground truth label: Positive	Ground truth label: Negative
	Positive	True positive (TP)	False positive (FP)
	Negative	False negative (FN)	True negative (TN)

General confusion matrix

Predicted label		Ground truth label: Has disease	Ground truth label: No disease
	Tested positive for disease	520	75
	Tested negative for disease	10	2850

Example for diagnostic test

Assessing Performance of a Model:

We can visualize the performance using a confusion matrix!

Step 13 in word document

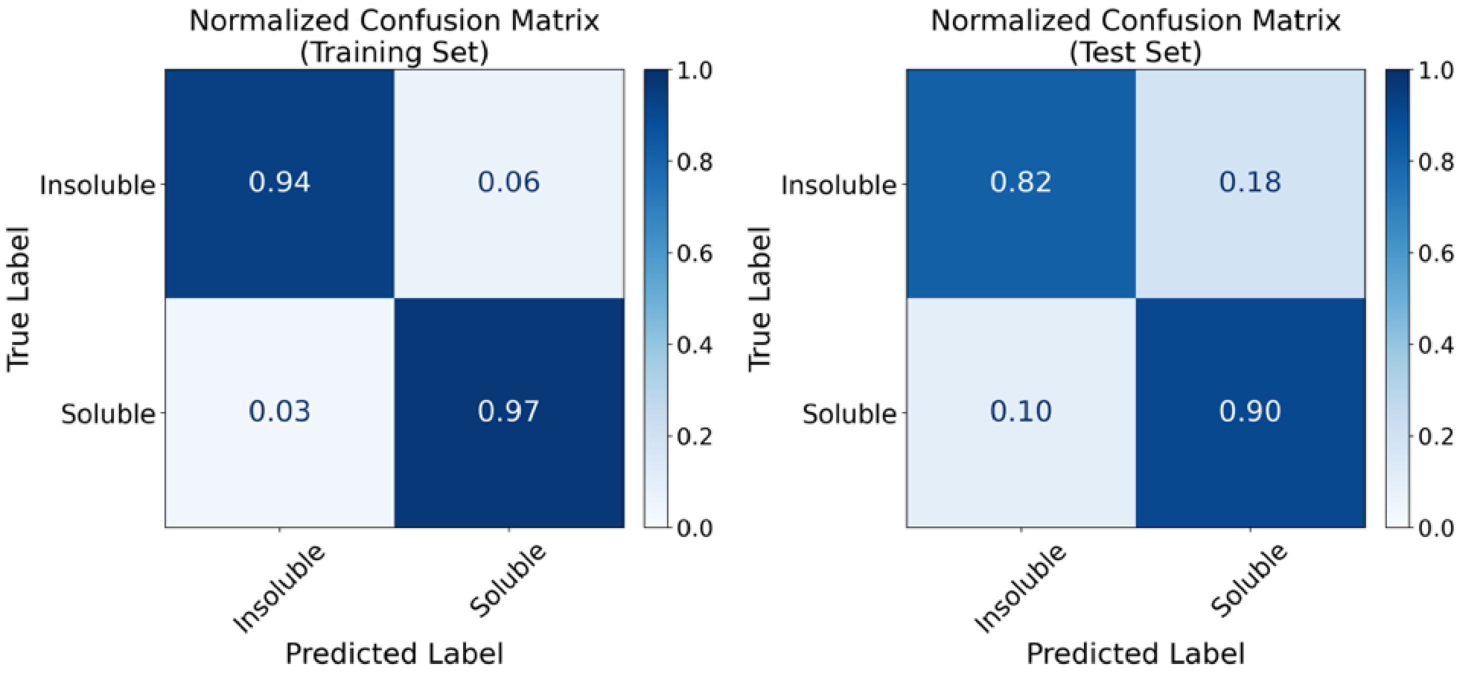

- Performance on training set is excellent
- Performance on the test set is worse

Test the parameters in the hold out and k-fold methods

- Run through step 14 (keep default in the one you are not testing)
  - Keep default parameters on step 12
- How do the test sizes (hold out) and number of folds (k-fold) affect
  - Accuracy, precision, recall?
  - Confusion matrix?

Topics for today

- Scaling features and outputs
- Cross validation methods
    - Train/test split
    - K-fold
- Evaluating model performance
    - Accuracy, precision, recall
    - Confusion matrix
- Decision tree classifier

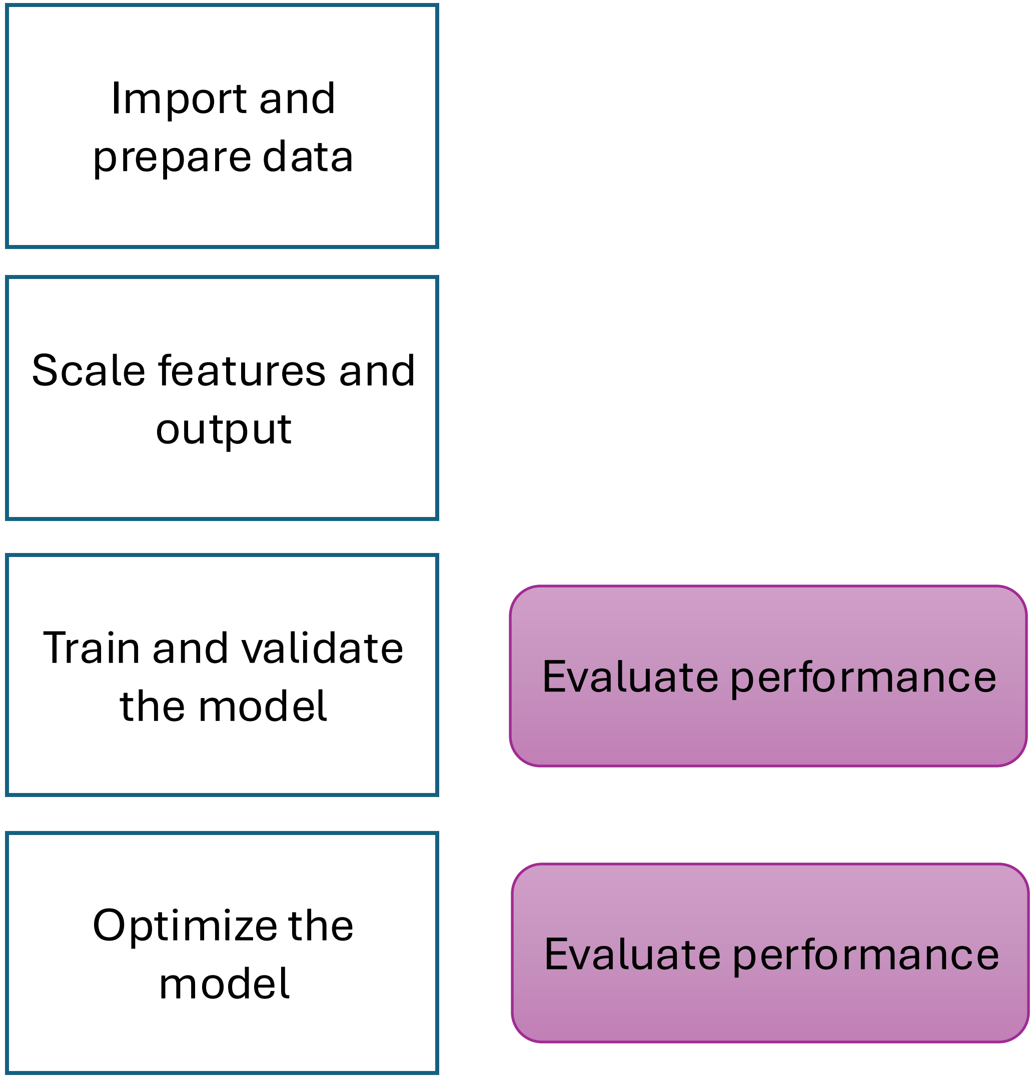

What type of machine learning model?

- **Supervised learning**: Input data is labeled -> label which input goes with which output
- **Unsupervised learning**: Input data is not labeled → model finds the patterns and relationships in the data

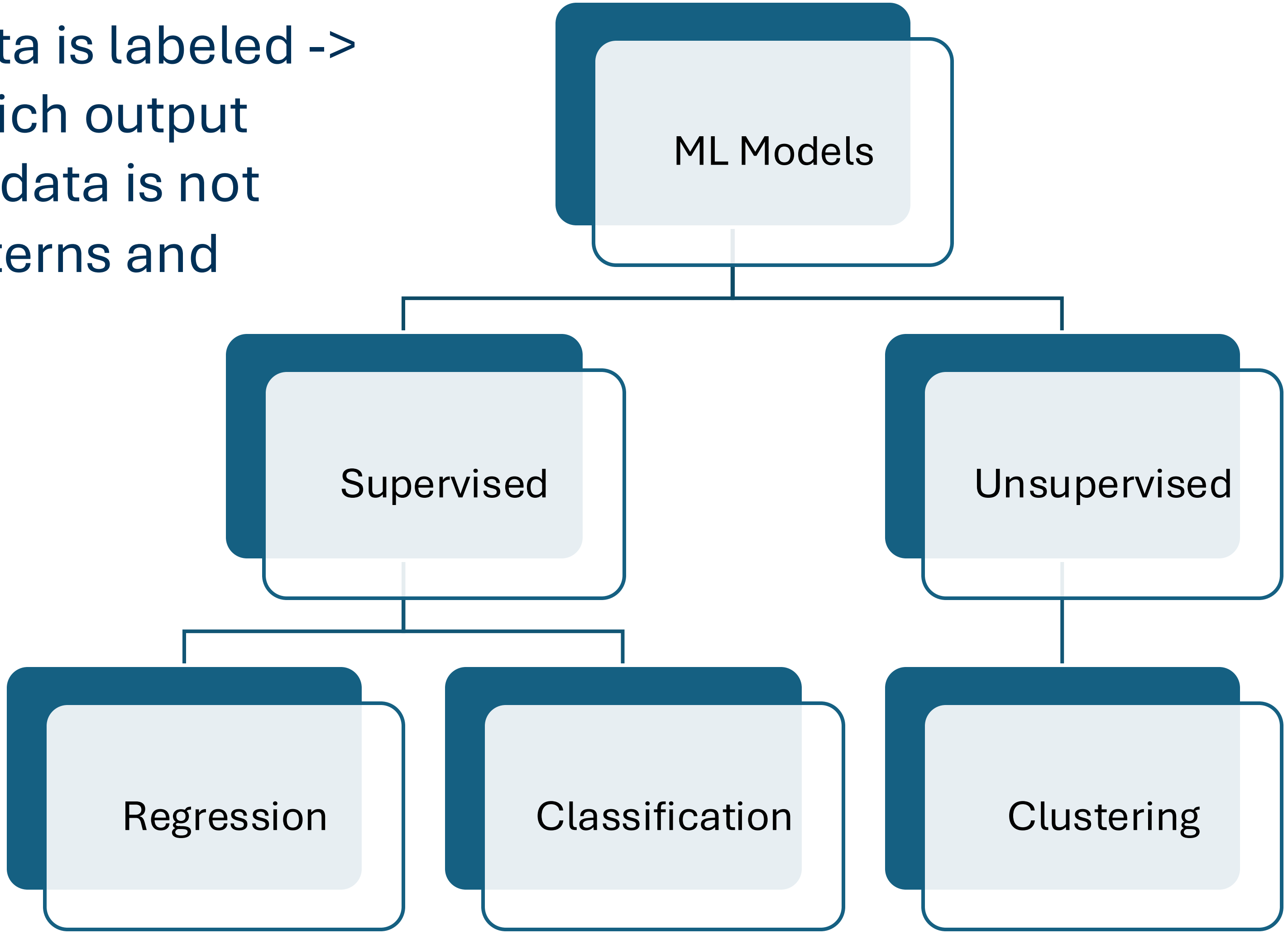

What Is a Decision Tree model?

- A decision tree is a type of supervised machine learning used to categorize or make predictions based on how a previous set of questions were answered.
- The model is a form of supervised learning, meaning that the model is trained and tested on a set of data that contains the desired categorization.
- Can be used for both regression and classification
- Decision trees imitate human thinking, so it’s generally easy for data scientists to understand and interpret the results.

Model learns a series of explicit if/then rules on feature values that result in a decision that predicts the target value

Decision tree example

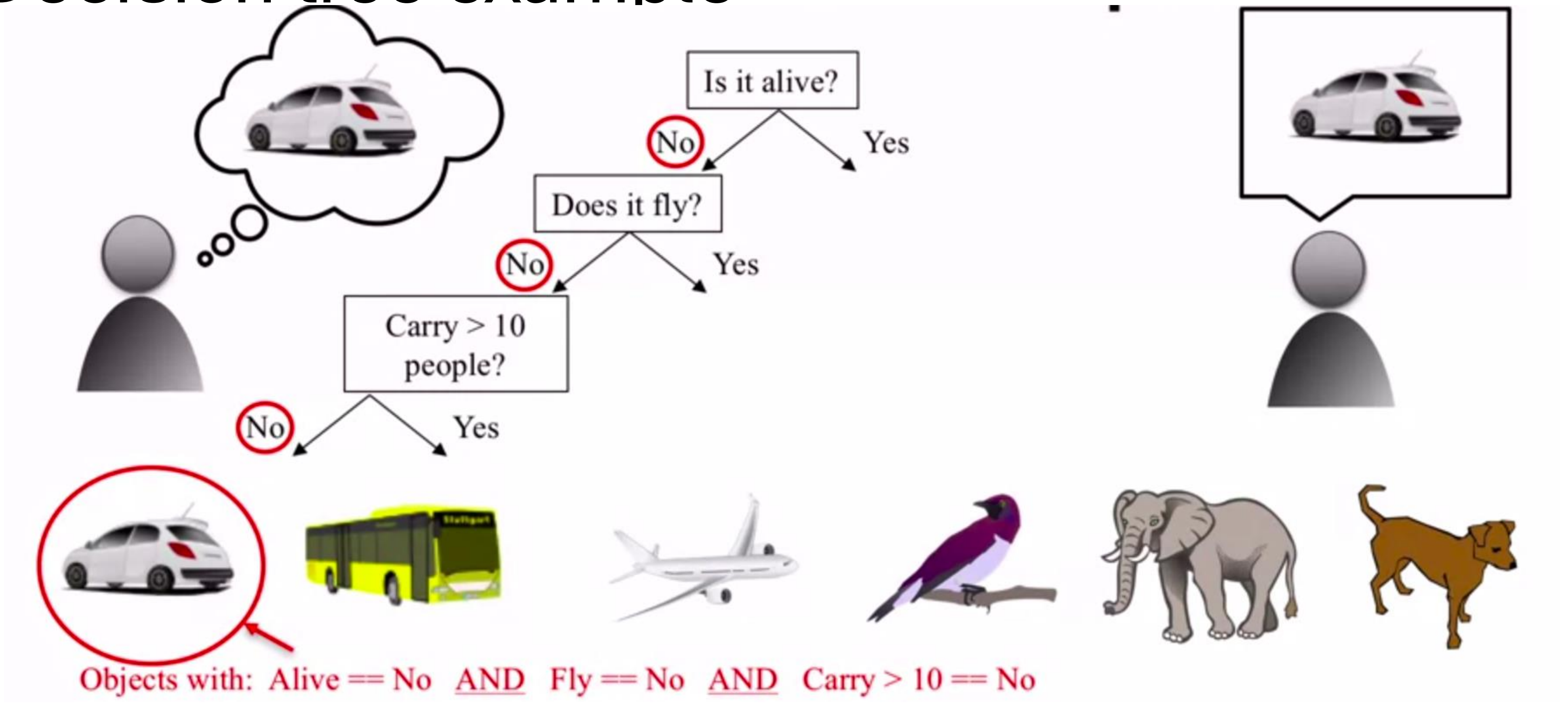

Figure from Volodymyr Kovenko 2020:
https://machine-learning-and-data-science-with-python.readthedocs.io/en/latest/assignment5_sup_ml.html

Random forest model

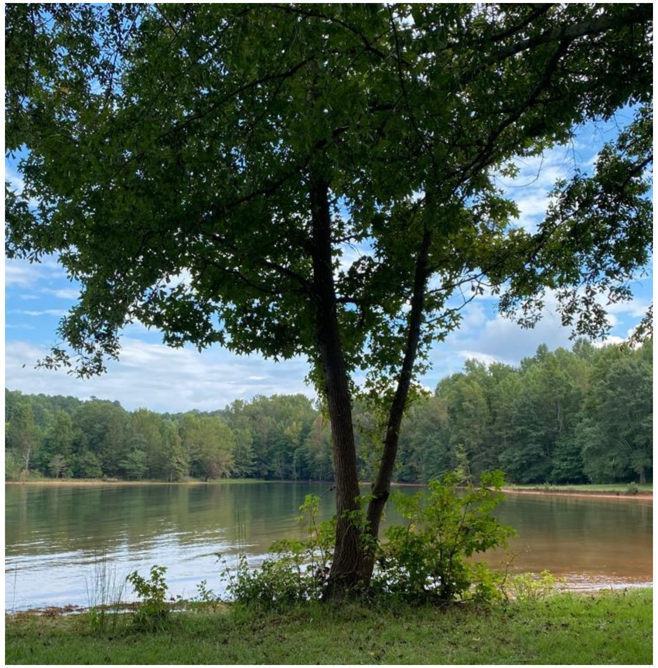

Decision tree – single person making the decisions

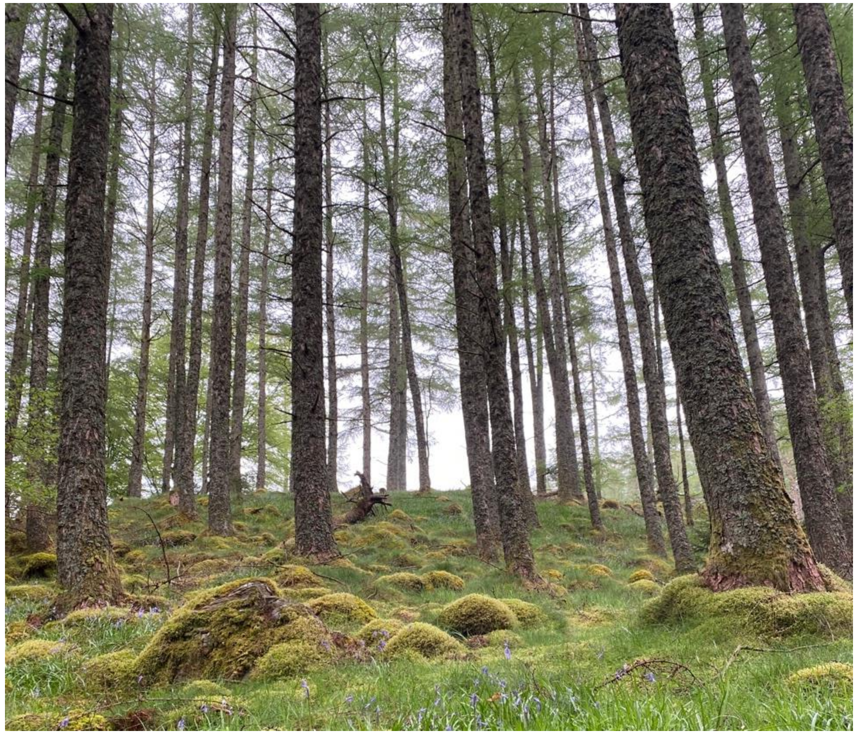

Random forest – multiple people making decisions

- A well-known machine learning model
- Random forest model is an ensemble of multiple learning models
- They combine to produce an aggregate model that is better than any one on its own
  - Averages out "mistakes" of any individual model

Advantages and Disadvantages of Decision Tree Model:

- **Advantages**
  - Intuitive interpretation of model
  - Able to predict highly non-linear boundaries
  - Well-suited for multi-class problems
- **Disadvantages**
  - Easily over-fit
  - Training can be expensive for large or high-dimensional data
  - Boundaries are discrete rather than continuous

Run decision tree classifier

Step 12 in word document

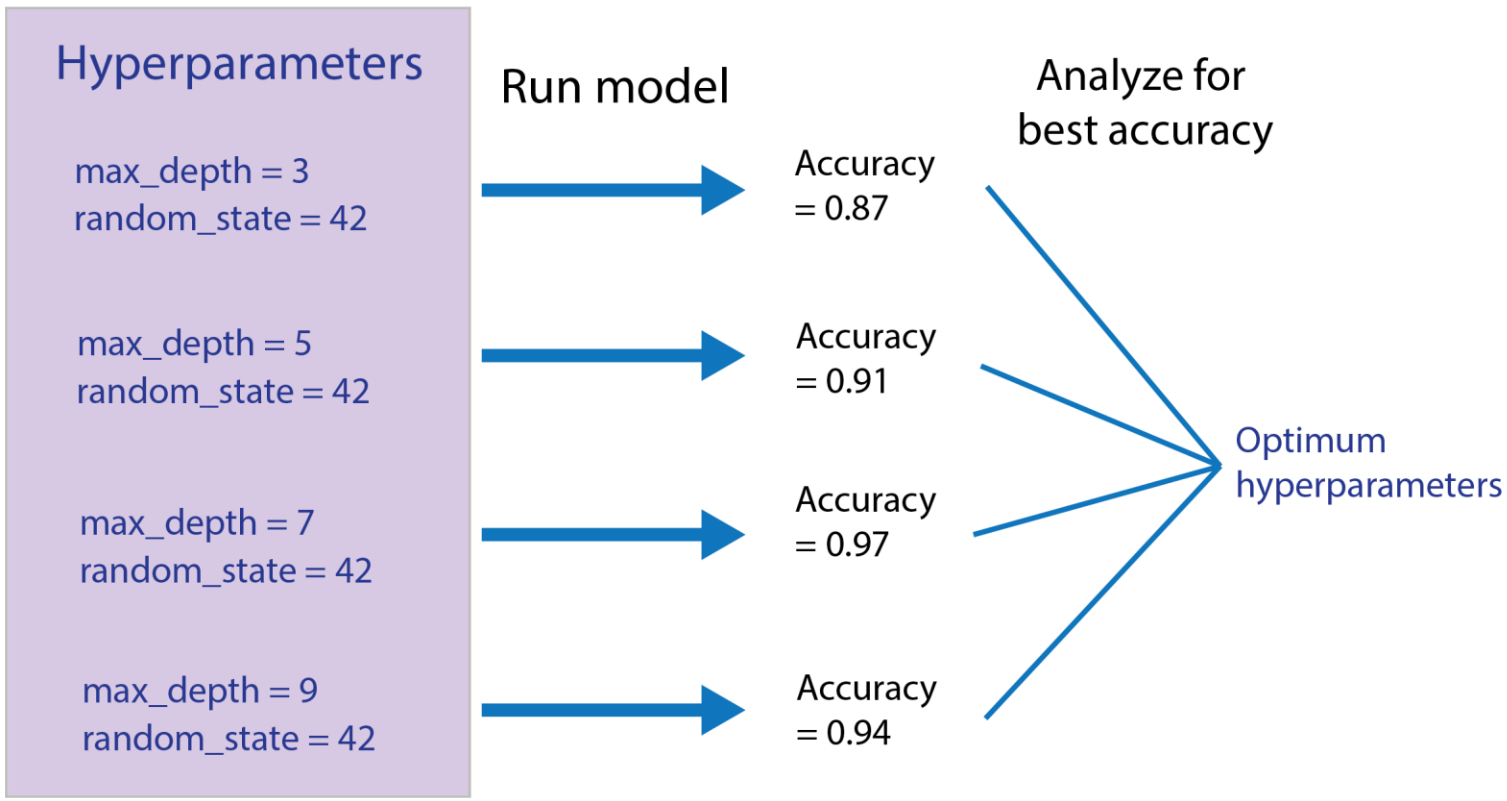

- You will run the decision tree classifier
- You will tune one hyperparameter (depth)
  - This is adjusting the model structure parameters to find one that gives the highest accuracy

Optimize the decision tree classifier

- Rather than testing the hyperparameters one by one by hand, you can optimize
- Step 15 in the code optimizes depth from 2 to 10
