# Supplementary material for: Introducing Data-Driven Materials Informatics into Undergraduate Courses through a Polymer Science Workshop
Source: J Chem Educ. 2025 Aug 15;102(9):3972–81. doi: 10.1021/acs.jchemed.5c00562 (PMC12424157; doi:10.1021/acs.jchemed.5c00562)
Supplement: Supplementary file 4 [file ed5c00562_si_004.docx]

**Supporting Information**

**Introducing data-driven materials informatics into undergraduate courses through a polymer science workshop**

Mona Amrihesari,^1^ Blair Brettmann^1,2*^

1. School of Chemical and Biomolecular Engineering, Georgia Institute of Technology, Atlanta, GA 30332
2. School of Material Science and Engineering, Georgia Institute of Technology, Atlanta, GA 30332

*corresponding author, blair.brettmann@chbe.gatech.edu

**Code for ML Solubility predictions**

**Content begins on next page**

**To run this code, follow these steps for easy access if you don’t have Jupyter notebook installed.**

Each blue box step should be inserted as a new block. Be sure to copy exactly from the blue boxes, including spaces and empty lines.

1. **Go to** [**https://colab.research.google.com**](https://colab.research.google.com)
2. **Press on “New Notebook” button**


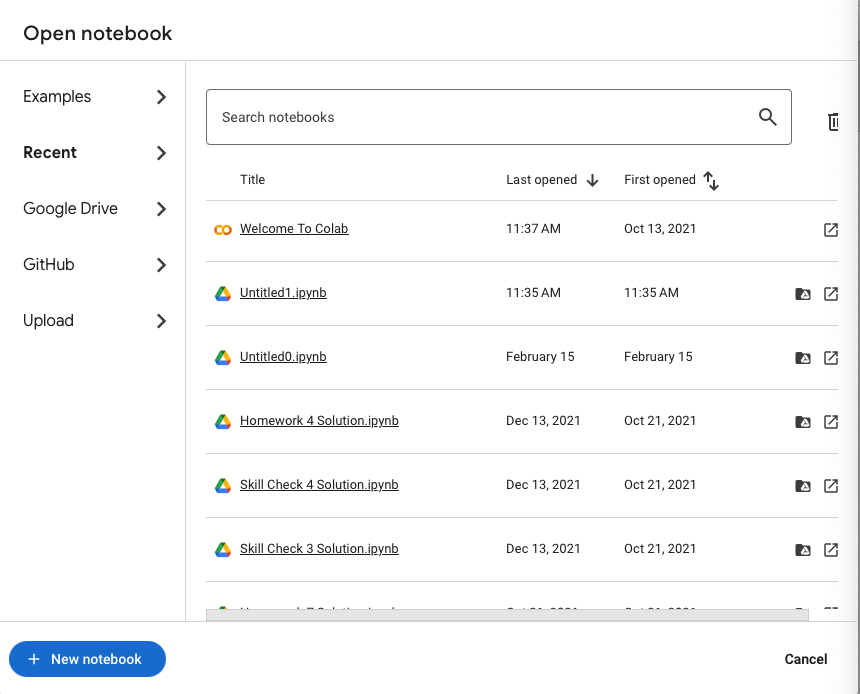


1. **Now, press on the files on the left sidebar and drag and drop the data file (Polymer _solvent_solubility.xlsx) as it is shown here.**

**
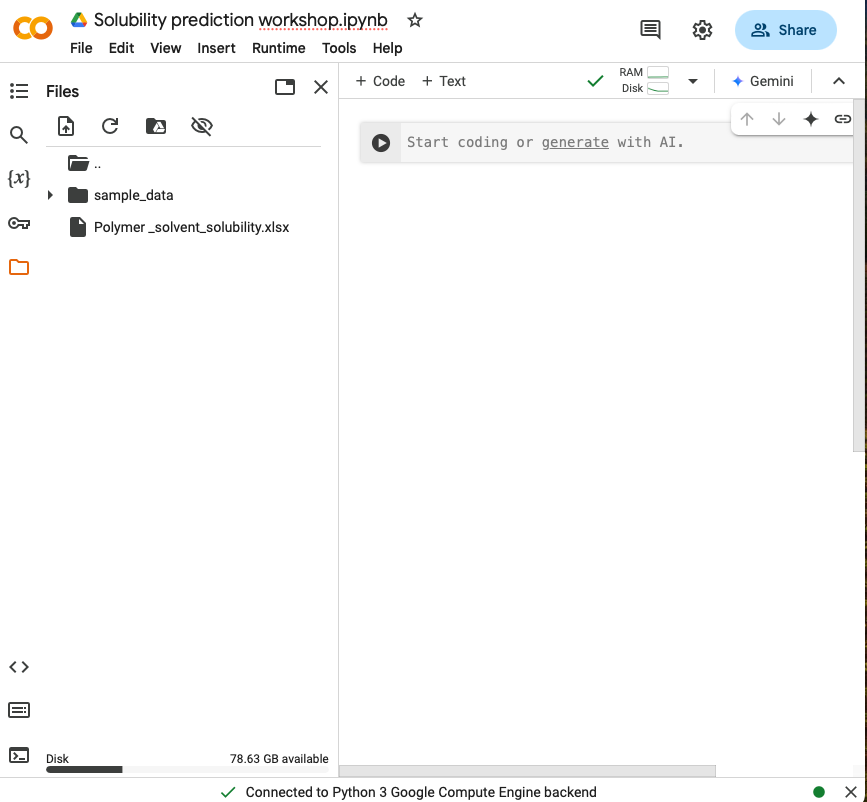
**

1. **Now, Let’s code! After you copy and paste the code for step 1 below, you will press the play button to run the code and you should see the printed message of “All packages are installed successfully!”**
2. **Now, you can press “+Code” button on the top to add another block for coding.**

**Code for ML solubility predictions**

1. First step in coding is always installing what we need. We will do that in two steps. First, installing all the required libraries, then importing the packages that we need. So, let’s use this function to install all the required libraries and check if it is successfully installed.

import subprocess

import sys

def install(package):

subprocess.check_call([sys.executable, "-m", "pip", "install", package])

required_packages = [

"pandas",

"numpy",

"matplotlib",

"scikit-learn",

"seaborn"

]

for package in required_packages:

install(package)

print("All packages installed successfully!")

1. We will import all the necessary packages using this code:

import pandas as pd

import numpy as np

import pylab as plt

from sklearn.preprocessing import StandardScaler

from sklearn.model_selection import train_test_split

from sklearn.model_selection import KFold

from sklearn.tree import DecisionTreeClassifier

from sklearn.metrics import accuracy_score

from sklearn.metrics import precision_score

from sklearn.metrics import recall_score

from sklearn.metrics import confusion_matrix, ConfusionMatrixDisplay

import matplotlib.pyplot as plt

import seaborn as sns

import matplotlib.pyplot as plt

print("All packages are installed and working!")

1. Next, we load the data that we need to analyze:

df = pd.read_excel('Polymer _solvent_solubility.xlsx')

1. We specify all the feature columns, starting from the third column in this dataset, as we will only work with numerical values. We can always print the results to verify everything!

column_label = df[df.columns[3:]].columns[:]

print(column_label)

1. We only need the values and not the headers, so let’s specify that. By printing the shape, we can check the number of rows and columns.

values = df[df.columns[3:]].values

print(values.shape)

1. Using the below code, we are deleting all the rows with empty values or NAN values. We will also set the x and y values. The y column is our prediction labels. Prediction labels are from -1 to 3 each corresponding to:

'-1 : solvent freeze',

'0 : insoluble',

'1 : partially soluble',

'2 : soluble',

'3 : solvent evaporated')

is_missing_value = np.array(df.isna())

valid_rows = []

for i in range(values.shape[0]):

if not any(is_missing_value[i,:]):

valid_rows.append(i)

X = np.array(values[valid_rows,:-1])

y = np.array(values[valid_rows,-1])

# Calculate the number of rows eliminated

total_rows = values.shape[0]

valid_rows_count = len(valid_rows)

eliminated_rows_count = total_rows - valid_rows_count

# Print the number of eliminated rows

print(f"Total rows in dataset: {total_rows}")

print(f"Valid rows retained: {valid_rows_count}")

print(f"Rows with missing values eliminated: {eliminated_rows_count}")

1. If you are interested in seeing how many instances are in each dataset, use the code below to plot them. If you do, you will notice that the data is imbalanced. There are different ways to handle imbalanced data, but for now, we will just remove the data that has low counts (next step).

bin_count = []

label_class = np.array([-1, 0, 1, 2, 3])

for label in label_class:

count = y[y == label].shape[0]

bin_count.append(count)

# Plot distribution

fig, ax = plt.subplots(figsize=(8, 5))

width = 0.35

bar_plot = ax.bar(label_class, bin_count, width)

# Set font size for labels, title, and ticks

ax.set_ylabel('Counts', fontsize=18)

ax.set_title('Distribution of Datapoints in Each Class', fontsize=20)

ax.set_xticks(label_class)

ax.set_xticklabels(('-1:solvent freeze', '0:insoluble', '1:partially soluble', '2:soluble', '3:solvent evaporated'), fontsize=15)

ax.tick_params(axis='y', labelsize=20) # Increase y-axis tick font size

# Rotate x-axis labels

plt.xticks(rotation=45)

# Show plot

plt.show()

1. Now, let’s delete the rows with two labels of “solvent freeze” and “solvent evaporated” and “partial soluble” class.

# Identify rows with missing values

is_missing_value = np.array(df.isna())

valid_rows = []

for i in range(values.shape[0]):

if not any(is_missing_value[i, :]): # Retain rows with no missing values

valid_rows.append(i)

# Extract valid rows (no missing values)

values_no_missing = values[valid_rows, :]

# Filter out rows where the prediction label (last column) is -1 or 3

y = np.array(values_no_missing[:, -1]) # Extract the prediction labels

valid_label_rows = (y != -1) & (y != 3) & (y != 1) # Create a mask to exclude solvent freeze (-1), solvent evaporated (3), and partially soluble (1) classes

# Apply the mask to get the filtered features and labels

X = np.array(values_no_missing[valid_label_rows, :-1]) # Features (all columns except the last)

y = np.array(values_no_missing[valid_label_rows, -1]) # Labels (last column)

# Statistics

total_rows = values.shape[0]

rows_after_missing_filter = values_no_missing.shape[0]

rows_after_label_filter = X.shape[0]

eliminated_rows_missing = total_rows - rows_after_missing_filter

eliminated_rows_label = rows_after_missing_filter - rows_after_label_filter

# Print the statistics

print(f"Total rows in dataset: {total_rows}")

print(f"Rows eliminated due to missing values: {eliminated_rows_missing}")

print(f"Rows eliminated due to Prediction Label (-1, 1 and 3): {eliminated_rows_label}")

print(f"Remaining rows: {rows_after_label_filter}")

1. Since the values are all different units and some models are sensitive to that, we need to scale the data prior to modeling.

ss = StandardScaler()

X_ss = ss.fit_transform(X)

y = y.astype(int)

1. We can use train/ test split function to divide the data into train and validation data with test size= 0.3 (This is adjustable). The validation set will be hidden from the model during training. You can split the data into test and train data using cross validation in the next block. **(Let’s try different test size= 0.2, 0.5, what do you hypothesize about the accuracy of the model?)**

X_train, x_validation, Y_train, y_validation = train_test_split(X_ss, y, test_size=0.3, random_state=42)

1. We use Kfold method to divide the data into train and test data. Also, we set the Kfold to runs for 5 iterations. In each iteration, it divides the data to train and test. It also shuffles the data. We use the standardized data for that.

(**Let’s try different Kfold, what do you hypothesize about the results? Remember in each fold, depend on the set number, one portion is test, and the rest are training sets**)

kf = KFold(n_splits=5, shuffle=True, random_state=42)

for train_index, test_index in kf.split(X_train):

x_train, x_test = X_train[train_index], X_train[test_index]

y_train, y_test = Y_train[train_index], Y_train[test_index]

1. Now let’s make some predictions and print them for the test set. We are checking for overall accuracy, precision and recall values. Each model has different set of hyperparameters which needs to be tuned. However, for now, we set the max_depth of the decision tree model to 5. **Try different depths (e.g., 3, 5, 7) and check the model accuracy using the accuracy, precision and recall, what do you hypothesize about the results?**

# Initialize and fit Decision Tree classifier

dt = DecisionTreeClassifier(random_state=42, max_depth=5)

dt.fit(x_train, y_train)

# Make predictions for train and test sets

y_train_pred = dt.predict(x_train)

y_test_pred = dt.predict(x_test)

# Calculate metrics with zero_division handling

accuracy = accuracy_score(y_test, y_test_pred)

# Since we have multiple labels (e.g., {0, 2}), specify the averaging method

precision = precision_score(y_test, y_test_pred, average="binary", pos_label=2, zero_division=0)

recall = recall_score(y_test, y_test_pred, average="binary", pos_label=2, zero_division=0)

# Print metrics

print("Accuracy:", accuracy)

print("Precision:", precision)

print("Recall:", recall)

1. You can print the results for the validation set too.

y_validation_pred = dt.predict(x_validation)

# Calculate metrics with zero_division handling

accuracy = accuracy_score(y_validation, y_validation_pred)

precision = precision_score(y_validation, y_validation_pred, average='weighted')

recall = recall_score(y_validation, y_validation_pred, average='weighted')

print("Accuracy:", accuracy)

print("Precision:", precision)

print("Recall:", recall)

1. Now, let’s visualize the results using confusion matrix for both train and test sets. We use normalized confusion matrix for easier comparison.

# Define updated class names

class_names = ["Insoluble", "Soluble"]

# Function to remove "Partially Soluble" class and keep only class labels {0, 2}

def filter_classes(y_true, y_pred):

mask = (y_true == 0) | (y_true == 2) # Keep only labels 0 and 2

return y_true[mask], y_pred[mask]

# Function to plot confusion matrices

def plot_confusion_matrices(y_train, y_train_pred, y_test, y_test_pred, y_validation, y_validation_pred):

# Filter out the "Partially Soluble" class (label 1)

y_train, y_train_pred = filter_classes(y_train, y_train_pred)

y_test, y_test_pred = filter_classes(y_test, y_test_pred)

y_validation, y_validation_pred = filter_classes(y_validation, y_validation_pred)

# Compute normalized confusion matrices with only labels {0, 2}

cm_train = confusion_matrix(y_train, y_train_pred, labels=[0, 2], normalize='true')

cm_test = confusion_matrix(y_test, y_test_pred, labels=[0, 2], normalize='true')

cm_validation = confusion_matrix(y_validation, y_validation_pred, labels=[0, 2], normalize='true')

# Set color bar range

vmin, vmax = 0, 1

# Create figure for training and test matrices

fig, axes = plt.subplots(1, 2, figsize=(16, 8), gridspec_kw={'width_ratios': [1, 1]})

# Function to plot confusion matrices with larger fonts and color bar matching size

def plot_cm(ax, cm, title):

disp = ConfusionMatrixDisplay(confusion_matrix=cm, display_labels=class_names)

disp.plot(ax=ax, cmap="Blues", xticks_rotation=45, values_format=".2f", colorbar=False)

# Increase annotation size

for text in ax.texts:

text.set_fontsize(20) # Make the existing numbers inside the confusion matrix larger

# The rest of the code is below:

# Increase tick label size

ax.set_xticklabels(class_names, fontsize=18)

ax.set_yticklabels(class_names, fontsize=18)

# Increase title and axis label sizes

ax.set_title(title, fontsize=20)

ax.set_xlabel("Predicted Label", fontsize=20)

ax.set_ylabel("True Label", fontsize=20)

# Set color limits for consistency

disp.im_.set_clim(vmin, vmax)

# Add a correctly sized color bar

cbar = fig.colorbar(disp.im_, ax=ax, fraction=0.046, pad=0.04)

cbar.ax.tick_params(labelsize=16) # Set font size for color bar labels

# Training set confusion matrix

plot_cm(axes[0], cm_train, "Normalized Confusion Matrix (Training Set)")

# Test set confusion matrix

plot_cm(axes[1], cm_test, "Normalized Confusion Matrix (Test Set)")

# Adjust layout and show

plt.tight_layout()

plt.show()

# Create a separate figure for validation matrix with color bar aligned

fig, ax = plt.subplots(figsize=(8, 7))

# Validation set confusion matrix

plot_cm(ax, cm_validation, "Normalized Confusion Matrix (Validation Set)")

# Show validation confusion matrix

plt.tight_layout()

plt.show()

# Example call to the function

plot_confusion_matrices(y_train, y_train_pred, y_test, y_test_pred, y_validation, y_validation_pred)

1. In this step, we are using the list of hyperparameter and a For loop to find the best max_depth for the Decision Tree Classifier model. The range is (2,10). After tuning the model, you can plot the confusion matrix and check the results.
   We then print the accuracy results.

# Define depth range

depths = np.arange(2, 10)

train_accuracies = np.zeros(len(depths))

test_accuracies = np.zeros(len(depths))

# Iterate over tree depths

for idx, depth in enumerate(depths):

# Train a Decision Tree model for the current depth

dt = DecisionTreeClassifier(max_depth=depth, random_state=42)

dt.fit(x_train, y_train)

# Evaluate accuracy on the training and test sets

train_accuracies[idx] = dt.score(x_train, y_train)

test_accuracies[idx] = dt.score(x_test, y_test)

# Determine the optimal depth and corresponding test accuracy

opt_test_accuracy = np.max(test_accuracies)

opt_index = np.argmax(test_accuracies)

opt_max_depth = depths[opt_index]

# Display results

print("Best model test accuracy:", opt_test_accuracy)

print("Optimum depth for Decision Tree:", opt_max_depth)

# Train the best Decision Tree model

best_dt = DecisionTreeClassifier(max_depth=opt_max_depth, random_state=42)

best_dt.fit(x_train, y_train)

# Make predictions

y_train_pred = best_dt.predict(x_train)

y_test_pred = best_dt.predict(x_test)

y_validation_pred = best_dt.predict(x_validation)

# Plot confusion matrices

plot_confusion_matrices(y_train, y_train_pred, y_test, y_test_pred, y_validation, y_validation_pred)
