# Supplementary material for: Introducing Data-Driven Materials Informatics into Undergraduate Courses through a Polymer Science Workshop
Source: J Chem Educ. 2025 Aug 15;102(9):3972–81. doi: 10.1021/acs.jchemed.5c00562 (PMC12424157; doi:10.1021/acs.jchemed.5c00562)
Supplement: Supplementary file 5 [file ed5c00562_si_005.pdf]

Supporting Information

Introducing data-driven materials informatics into undergraduate courses through a polymer science workshop

Mona Amrihesari,[1] Blair Brettmann[1,2*]

1) School of Chemical and Biomolecular Engineering, Georgia Institute of Technology, Atlanta, GA 30332
2) School of Material Science and Engineering, Georgia Institute of Technology, Atlanta, GA 30332

*corresponding author, blair.brettmann@chbe.gatech.edu

Guided activity sheet

Content begins on next page

Materials informatics for polymer property prediction in-class activity

Purpose:

1. Introduce the following basic concepts in machine learning/data science
    a. Preparing a dataset for machine learning
    b. Training the dataset using train/test split and Kfold methods
    c. Hyperparameter tuning with a decision tree classifier model
    d. Analyzing prediction quality with respect to accuracy, precision and recall, in particular through the confusion matrix
2. See how machine learning/data science can be used in polymer property prediction

Resource: Polymer solubility dataset (Polymer_solvent_solubility.xlsx), Google Colab (collab.research.google.com)

Product of the activity: Each student must turn in answers to the questions on the day of the activity in class. You may work in a group and all group members should turn in the same document.

Questions to answer (25 pts each)

1. Provide the accuracy, precision and recall for a test/train split with test sizes of 0.2, 0.3, 0.5. How does the performance of the model change with the test size?
2. Provide the accuracy, precision and recall for a model trained with the KFold method with 5 iterations and two other numbers of iterations of your choice. What do you conclude about how the number of iterations impacts the model? performance? What do you conclude about the test/train split vs. KFold methods?
3. Provide the accuracy, precision and recall for a model trained with a method of your choice and with hyperparameter tuning at different depths (3, 5, 7). How does the depth impact the accuracy, precision and recall of the model? How do these compare to the optimum depth found in step 5?
4. Provide the normalized confusion matrix for the training and test sets. Which has a better performance? How did you determine which has the better performance?
